# Evaluating the clinical effects of GLP-1 receptor agonists for Alzheimer's and Parkinson's diseases using minimal clinically important difference: systematic review and meta-analysis

**DOI:** 10.1007/s12272-026-01615-y

**Published:** 2026-05-07

**Authors:** Yomna Elghanam, Eunyoung Kim

**Affiliations:** 1 Data Science, Evidence-Based and Clinical Research Laboratory, Department of Health, Social, and Clinical Pharmacy, College of Pharmacy, Chung-Ang University, Seoul, 06974 Republic of Korea; 2 The Graduate School for Pharmaceutical Industry Management, College of Pharmacy, Chung-Ang University, Seoul, 06974 Republic of Korea; 3 Department of Pharmaceutical Regulatory Science, College of Pharmacy, Chung-Ang University, Seoul, 06974 Republic of Korea; 4 Evidence-Based and Clinical Research Laboratory, Department of Health, Social, and Clinical Pharmacy, College of Pharmacy, Chung-Ang University, 84 Heukseok-ro, Dongjak-gu, Seoul, 06974 Republic of Korea

**Keywords:** Alzheimer’s disease, Parkinson’s disease, Mild cognitive impairment, Glucagon-like peptide-1 receptor agonist, Cognition, Disease-modification

## Abstract

Glucagon-like peptide-1 receptor agonists (GLP-1RAs) are promising candidates for Alzheimer’s disease (AD) and Parkinson’s disease (PD). However, their effects in non-diabetic populations, independent of metabolic confounding, remain unclear. We evaluated the effects of GLP-1RAs on cognition, clinical outcomes, biomarkers, and safety in non-diabetic individuals with PD, AD, and mild cognitive impairment. We assessed the clinical meaningfulness of these effects using minimal clinically important difference thresholds. Relevant studies were retrieved from PubMed, Embase, and Web of Science from inception to November 2025. A random-effects meta-analysis was applied to calculate standardized mean differences (SMDs), mean differences (MDs), and risk ratios with 95% confidence intervals (CIs). The protocol was registered in PROSPERO (CRD420261277032). Fourteen randomized controlled trials enrolling 1260 participants were included. GLP-1RAs showed a small statistically significant improvement in global cognition (SMD 0.14, 95% CI 0.01 to 0.27; I^2^ = 7%), supported by high-certainty evidence. Despite statistical significance, findings suggest only a trivial probability (1%) of a clinically important benefit. Conversely, GLP-1RAs were associated with poorer verbal fluency (SMD − 0.43, 95% CI − 0.79 to − 0.08; I^2^ = 0%), supported by high-certainty evidence. For clinical severity, function, depression, and PD-related outcomes, pooled estimates generally favored GLP-1RAs, but none reached statistical significance. A significant between-disease subgroup difference was observed for function. In the PD subgroup, GLP-1RAs significantly improved depression symptoms relative to control (MD − 2.09, 95% CI − 3.99 to − 0.20; I^2^ = 0%). Nevertheless, this magnitude of improvement remained below the threshold for clinically important benefit. Biomarker findings were inconsistent across trials. GLP-1RAs significantly reduced weight and were associated with poorer tolerability and increased gastrointestinal adverse events. Current evidence provides no convincing support for a clinically meaningful or disease-modifying effect of GLP-1RAs, and adverse effects may limit their clinical utility. Large-scale trials are needed to definitively weigh potential benefits against associated risks.

**Supplementary Information:**

The online version contains supplementary material available at 10.1007/s12272-026-01615-y.

## Introduction

Alzheimer’s disease (AD) and Parkinson’s disease (PD) are progressive neurodegenerative disorders driven by complex, interconnected cellular and molecular processes (Elghanam et al. 2024; Ibrahim et al. 2025). Recently, glucagon-like peptide-1 receptor agonists (GLP-1RAs) have attracted increasing interest as potential therapeutic agents for neurodegenerative diseases. GLP-1RAs are incretin-based therapies that activate the GLP-1 receptor to enhance glucose-dependent insulin secretion, suppress inappropriately elevated glucagon secretion, slow gastric emptying, and reduce appetite (Zheng et al. 2024). GLP-1RAs are approved by the US Food and Drug Administration for glycemic control in type 2 diabetes mellitus (T2DM) and for chronic weight management (Nauck et al. 2021; Zheng et al. 2024). Although developed for metabolic indications, GLP-1RAs have been evaluated for potential associations with neurocognitive outcomes. A recent study reported a significantly lower risk of all-cause dementia or cognitive impairment with GLP-1RA exposure (Seminer et al. 2025).

This emerging clinical signal is biologically plausible, as GLP-1 receptors are expressed in the central nervous system (CNS) and GLP-1RA signaling may exert direct neuroprotective effects (Zheng et al. 2024). In preclinical AD models, GLP-1 (7–36) amide has been associated with reduced neuroinflammatory signaling, including interleukin-1β, alongside attenuated microglial activation and reduced amyloid-related pathology. It has also been linked to improved synaptic plasticity and cognition, as well as enhanced mitochondrial function. Moreover, it may support learning and memory by facilitating long-term potentiation (Hölscher 2010; Egecioglu et al. 2013). Other studies have proposed a neuroprotective role for GLP-1 analogs as they reduce oxidative stress and apoptosis (García-Casares et al. 2023). In PD, GLP-1RAs have been proposed to help preserve dopaminergic neuronal function through neuroprotective GLP-1 signaling (Zheng et al. 2024).

Prior research on GLP-1RAs has largely focused on the diabetic population, where cognitive impairment is closely intertwined with hypoglycemia, insulin resistance, and diabetes-related complications (Luan et al. 2022; Tang et al. 2023; García-Casares et al. 2023; Kuate Defo et al. 2024). One systematic review (SR) reported that exenatide, dulaglutide, and liraglutide improved general cognition in the T2DM population (García-Casares et al. 2023). However, it remains unclear whether these observed effects primarily reflect indirect benefits through improved glycemic control and cardiovascular risk profiles, or disease-specific neuroprotective effects. Recent research has evaluated the effects of GLP-1RAs on cognition in AD; however, conclusions remain constrained by a narrow outcome scope, limited randomized evidence, and the pooling of cognitively distinct measures under broad constructs, which may complicate construct validity and clinical interpretability (O’Mara et al. 2026). These limitations underscore the need for methodologically robust analyses in non-diabetic cohorts to determine whether GLP-1RAs exert clinically relevant neuroprotective effects independent of diabetes-related confounding.

Although AD, PD, and mild cognitive impairment (MCI) are clinically distinct, they share downstream pathways of neuronal injury linked to metabolic dysfunction. GLP-1RAs may offer benefits across these heterogeneous conditions by modulating shared mechanisms (Athauda et al. 2026). Consistent with a prior meta-analysis (MA) that pooled AD, PD, and dementia with Lewy bodies cohorts to examine the effects of cholinesterase inhibitors (D’Angremont et al. 2023), our study synthesized evidence from randomized controlled trials (RCTs) of GLP-1RAs in non-diabetic adults with AD, PD, or MCI. The primary outcome was global cognitive performance. Secondary outcomes included cognitive subdomains, function, clinical severity, PD-related outcomes, depression, and available biomarker measurements. The safety and tolerability of GLP-1RAs were also characterized. In addition, potential differences in treatment effects according to disease population, GLP-1RA agent, geographic region, and trial duration were assessed. A minimal clinically important difference (MCID) framework was applied to determine whether observed changes were clinically meaningful, thereby extending the interpretation of findings beyond statistical significance alone.

## Materials and methods

The protocol for this SR and MA was preregistered in the International Prospective Register of Systematic Reviews (PROSPERO; CRD420261277032) and followed the Preferred Reporting Items for Systematic Reviews and Meta-Analyses 2020 (PRISMA) guidelines (Table S1; Page et al. 2021).

### Search method and study selection

A comprehensive electronic search was conducted across three databases, PubMed, Embase, and Web of Science, from inception to November 2025. The search terms included: “glucagon-like peptide-1 receptor agonist*”, “semaglutide”, “liraglutide”, “exenatide”, “dulaglutide”, “albiglutide”, “lixisenatide”, “Dementia”, “Alzheimer disease” [MeSH Terms], “vascular dementia”, “mild cognitive impairment”, and “Parkinson disease” [MeSH Terms]. The detailed search strategies are provided in Table S2. In addition, we searched ClinicalTrials.gov to identify ongoing or unpublished studies, screened conference abstracts and proceedings for potentially relevant reports, and performed backward and forward citation searching.

### Eligibility criteria

The eligibility criteria were set according to the PICOS (Population, Intervention, Comparison, Outcome, and Study Design) framework.

#### Population

Adults with cognitive impairment within the dementia spectrum, including AD, vascular dementia, PD dementia, and Lewy body-related dementias, as well as adults with MCI or cognitive decline, were eligible for inclusion. Disorders in which cognition was a core outcome were prioritized, and MCI was considered a pre-dementia stage. Eligible trials were required to enroll non-diabetic participants, defined as those with no diagnosis of type 1 diabetes mellitus (T1DM) or T2DM at baseline. No restrictions were applied regarding language, publication date, setting, or follow-up duration.

#### Intervention and comparator

Treatment with a GLP-1RA at any dose, route, or regimen was considered eligible. Placebo, standard care, or usual care was accepted as the comparator, and background therapies were permitted provided that co-interventions were balanced between groups.

#### Outcomes

The primary outcome was global cognition assessed using validated global cognitive measures. The secondary outcomes included domain-specific cognitive performance, functional outcomes, global clinical severity, PD-specific motor and non-motor symptom severity, neuropsychiatric outcomes (including depression and mood), quality of life (QoL), safety, and tolerability. Relevant biomarkers and neuroimaging endpoints were also identified.

#### Study design

RCTs were eligible for inclusion. Post hoc analyses of eligible RCTs were included in the qualitative synthesis to capture additional outcomes (e.g., subgroup effects, biomarker endpoints, or longer-term follow-up). To avoid duplicating participants, data from post hoc reports were not pooled separately in the quantitative MA when overlapping with the parent trial dataset, and the primary trial report was prioritized for effect estimation.

### Exclusion criteria

The exclusion criteria included non-randomized study designs, animal or in vitro studies, reviews, editorials, letters, protocols, and conference abstracts. Trials enrolling participants with T1DM, T2DM, or mixed diabetic and non-diabetic samples for which non-diabetic results could not be extracted separately, were excluded.

### Data screening and extraction

Two authors independently screened titles and abstracts for eligibility, followed by a full-text assessment of potentially relevant records. Data were extracted independently by two authors. Information on study identifiers, design characteristics (trial registration number, country, trial phase, and funding), population characteristics (disease, diagnostic criteria, non-diabetic status, sample size, and analyzed population), and intervention details (route, dose, frequency, and treatment duration) was extracted. Baseline characteristics were extracted for each arm. For quantitative synthesis, outcome data were extracted for each treatment arm, including the time point, outcome scale, mean change from baseline with standard deviations (SDs), and corresponding sample sizes. Disagreements at any stage were resolved by consensus.

### Outcome definitions

#### Primary outcome

For each trial, a single primary global cognitive outcome was selected as the main prespecified endpoint for the primary MA. For the AD and MCI populations, the following hierarchy was applied. Alzheimer’s Disease Assessment Scale-Cognitive Subscale (ADAS-Cog) was prioritized as it is one of the most established and widely used cognitive outcome measures in AD trials and has long been a mainstay for tracking cognitive change over time (Levine et al. 2020). Alzheimer’s Disease Assessment Scale-Executive composite was ranked next as it was specifically developed for MCI populations to improve sensitivity to longitudinal change by supplementing the ADAS-Cog framework with executive function measures (Jacobs et al. 2020). Mini-Mental State Examination (MMSE) was ranked lower as it is widely used for cognitive screening but may be less suitable as the preferred primary endpoint when more detailed AD-focused measures are available (Levine et al. 2020). Wechsler Memory Scale total score was placed last as it was reported less consistently across the included trials, appearing only in one trial (Gejl et al. 2016; Dzikon 2020). For PD populations, Mattis Dementia Rating Scale-Second Edition (MDRS-2) total score was prioritized as it is a broader and more comprehensive measure of global cognition (Matteau et al. 2012). Montreal Cognitive Assessment (MoCA) was placed second as it remains fundamentally a brief screening instrument rather than a detailed global cognitive measure (Skorvanek et al. 2018). Scales for Outcomes in Parkinson’s Disease-Cognition was placed third because, compared with MDRS-2 and MoCA, it has been less extensively adopted and has been classified as recommended with caveats, partly because of limited data on sensitivity to change, suggesting it may not be a suitable instrument for treatment trials (Skorvanek et al. 2018). When multiple eligible global cognitive measures were reported within a trial, the highest-ranked measure was selected as the primary outcome, and the remaining global cognitive measures were examined in sensitivity analyses.

#### Secondary outcomes

Secondary cognitive outcomes were grouped a priori into the memory, executive/language, and attention/processing speed domains. Verbal fluency tests (semantic/category or phonemic/letter fluency) were classified within the executive/language domain (Shao et al 2014). When more than one fluency test was reported, a single prespecified measure was selected to ensure that each study contributed only one effect size per domain (semantic/category fluency preferred, followed by total fluency, and then phonemic/letter fluency). Semantic fluency was prioritized because it is often more strongly affected than phonemic/letter fluency in disorders such as AD (Kwak et al. 2022). Moreover, PD patients with semantic fluency deficits are more susceptible to dementia (Yang et al. 2022).

Functional outcomes were defined as measurements of everyday functioning, including activities of daily living, instrumental activities of daily living, functional independence, and global functional status. Eligible functional measures included the Alzheimer’s Disease Cooperative Study-Activities of Daily Living (ADCS-ADL; Chandler et al. 2025), Instrumental Activities of Daily Living Scale (Guo and Sapra 2022), and Schwab and England Activities of Daily Living Scale (Choi et al. 2017). In PD trials, the Movement Disorder Society-Sponsored Revision of the Unified Parkinson’s Disease Rating Scale Part II (MDS-UPDRS Part II), which measures motor experiences of daily living, was treated as a functional outcome (Goetz et al. 2008).

Global clinical severity and progression outcomes were defined as clinician- or patient-rated measures intended to capture the overall disease severity or change over time. These included the Clinical Dementia Rating-Sum of Boxes (CDR-SoB; Tzeng et al. 2022) and global impression measures, such as the Clinical Global Impression of Severity (Forkmann et al. 2011) and the Patient Global Impression of Severity (Martínez-Martín et al. 2016), when reported. In PD trials, composite MDS-UPDRS global scores (Parts I–III) were also treated as a global measure of the overall motor and non-motor disease burden.

For PD-specific clinical outcomes, motor severity was defined using clinician-rated motor examination measures, primarily the MDS-UPDRS Part III, and non-motor symptom severity was defined using validated non-motor symptom instruments, including the MDS-UPDRS Part I and the Non-Motor Symptoms Scale (NMSS; Goetz et al. 2008; van Wamelen et al. 2021). In addition, motor complications were captured using the MDS-UPDRS Part IV (Goetz et al. 2008). When motor severity was reported in both on-medication and off-medication states, data were extracted and analyzed separately by state. On-medication assessments were defined as evaluations of motor impairment assessed under optimal dopaminergic treatment. Off-medication assessments were defined as those performed after withholding dopaminergic therapy for a prespecified washout period, commonly overnight. Assessments performed after additional investigational drug washout were recorded explicitly as distinct endpoints.

Mood and depression outcomes were assessed using validated scales, including the Montgomery–Åsberg Depression Rating Scale (MADRS), Geriatric Depression Scale, and Patient Health Questionnaire-9 (PHQ-9; Williams et al. 2012). QoL outcomes were assessed using the Parkinson’s Disease Questionnaire-39 Summary Index (PDQ-39 SI; Morley et al. 2015).

Neurodegeneration-related biomarkers and neuroimaging outcomes were extracted when reported, including amyloid-β (Aβ) measures in plasma and cerebrospinal fluid (CSF), and metabolic/neuroimaging indices such as fluorodeoxyglucose positron emission tomography (FDG-PET) measures of cerebral glucose metabolism. In PD trials, dopaminergic imaging biomarkers were extracted, including dopamine transporter single-photon emission computed tomography (DaT-SPECT). It quantifies striatal dopamine transporter binding as a marker of presynaptic nigrostriatal terminal integrity. Anthropometric outcomes relevant to GLP-1RAs, including body weight (kg) and body mass index (BMI, kg/m^2^), were also extracted.

To ensure consistent interpretation across scales, the directionality of each instrument was prespecified based on its original scoring. For measures in which higher scores indicate better performance or greater independence (e.g., MoCA, MMSE, MDRS-2, and ADCS-ADL), higher values represent better outcomes. For measures in which higher scores indicate worse impairment or symptom burden (e.g., CDR-SoB, MDS-UPDRS Parts I–IV, NMSS, MADRS, PHQ-9, and PDQ-39 SI), higher values represent worse outcomes. Since higher ADAS-Cog scores indicate worse cognition, its direction was inverted (multiplied by − 1) before pooling with other global cognition measures so that higher values consistently reflected better cognitive performance.

Safety outcomes included adverse events (AEs) of any type, such as gastrointestinal and neuropsychiatric AEs, injury-related events, and hypoglycemia. Tolerability was assessed primarily based on discontinuation due to AEs.

### Risk of bias and certainty of the evidence

The risk of bias (RoB) was independently evaluated by two authors using the Cochrane RoB 2 tool (Sterne et al. 2019). The certainty of evidence for each outcome was appraised using the Grading of Recommendations, Assessment, Development, and Evaluation (GRADE) framework across five domains, RoB, inconsistency, indirectness, imprecision, and other considerations, and rated as high, moderate, low, or very low (Balshem et al. 2011). Disagreements were resolved through consensus among the authors.

### Data analysis

MAs were conducted using Review Manager (RevMan) version 5.4.1. When at least 10 studies were available, publication bias was assessed using Egger’s regression test implemented in R software (version 4.5.1; R Core Team, Vienna, Austria; https://www.r-project.org). For continuous outcomes, treatment effects were summarized as standardized mean differences (SMDs; Hedges’ g) with 95% confidence intervals (CIs) when conceptually similar constructs were assessed using different instruments or scales (Deeks et al. 2024). When outcomes were measured using the same scale across studies, treatment effects were pooled using the mean difference (MD) with 95% CIs. For dichotomous outcomes, effects were summarized as risk ratios (RRs) and absolute effects with 95% CIs. Studies reporting multiple eligible intervention arms were combined using RevMan software for analysis as a single pairwise comparison.

When outcomes were reported as standard errors (SEs), SDs were obtained from the SE of the mean by multiplying by the square root of the sample size: SD = SE × √n. When SDs for changes from baseline were not reported, they were derived from baseline and endpoint SDs using the standard formula recommended in the Cochrane Handbook, which requires an assumed or externally estimated baseline–endpoint correlation (r) (Higgins et al. 2024). When the correlation was not available from trial reports, a baseline–endpoint correlation of r = 0.7 was assumed as a conservative estimate (Higgins et al. 2024). This approach has been used in a previously published MA when correlations between time points were unavailable (Kawaguchi et al. 2021).

Pooled estimates were calculated using a random-effects model to account for variation across studies. Statistical heterogeneity was assessed using the χ^2^ test (p < 0.10 indicating statistically significant heterogeneity) and quantified using I^2^. Percentages of I^2^ that were around 25%, 50%, and 75% indicated low, moderate, and high heterogeneity, respectively. A two-sided p ≤ 0.05 was considered statistically significant for overall pooled effects.

Meta-regression was conducted using a random-effects framework to examine whether the treatment effect varied as a function of treatment duration or disease group. This analysis was performed only when a sufficient number of studies contributed data to the outcome to support a single-moderator model and reduce the risk of overfitting (Rao et al. 2017).

Sensitivity analyses were conducted to assess the robustness of the primary findings by (i) excluding studies judged to be at a high RoB; (ii) excluding trials that enrolled prediabetic patients; (iii) repeating the primary cognitive analysis using alternative global cognitive scale selection when multiple measures were reported within a trial, with the same procedure applied to functional outcomes; and (iv) performing a leave-one-out analysis for the primary outcome, whereby the MA was iteratively repeated after omitting one study at a time to evaluate the influence of individual trials on the pooled effect estimate.

### Minimal clinically important difference

As an exploratory analysis for outcomes pooled using different measurement scales, we prespecified a standardized minimal important difference (MID) of 0.30 SD units (and the corresponding direction for improvement for each outcome) as a clinically meaningful threshold. This distribution-based threshold was selected based on methodological reviews on health-related QoL and patient-reported outcome instruments. These reviews identified the 0.30 and 0.50 SD thresholds as the most frequently reported and accepted MIDs when anchor-based values are unavailable or heterogeneous across instruments (Ousmen et al. 2018; Mouelhi et al. 2020). As a sensitivity analysis, we also evaluated a more conservative MID of 0.50 SD units to assess the robustness of our clinical inferences.

MCID-based analyses for ADAS-Cog, MMSE, and CDR-SoB were also considered exploratory. Thresholds were selected based on previously reported anchor-based MCID estimates for clinically meaningful decline in early-stage AD. MCID thresholds in the direction of improvement have not been established in the published literature (Hamilton et al. 2025). The corresponding opposite-direction thresholds were applied pragmatically to interpret treatment benefit, consistent with a previous study (Hsu et al. 2025). Thresholds for clinically meaningful improvement and decline were set at − 2 and 2 points for ADAS-Cog, respectively; 1 and − 1 points for MMSE; and − 1 and 1 points for CDR-SoB (Hamilton et al. 2025; Hsu et al. 2025).

For the PD-related outcomes, published MCID thresholds were applied to the original scales. For MDS-UPDRS Part I, the thresholds for improvement and decline were − 2.64 and 2.45 points, respectively, and for MDS-UPDRS Part II they were − 3.05 and 2.51 points (Horváth et al. 2017a). For MDS-UPDRS Part III, the corresponding thresholds were − 3.25 and 4.63 points (Horváth et al. 2015). For MDS-UPDRS Part IV, the corresponding thresholds were − 0.9 and 0.8 points (Makkos et al. 2019). For the PDQ-39 SI, the thresholds for improvement and decline were − 4.72 and 4.22 points, respectively (Horváth et al. 2017b). For MADRS, an MCID threshold for improvement of − 4.94 points was identified, whereas a corresponding threshold for decline was not identified (Hori et al. 2025). The selected thresholds were derived using anchor-based methods supplemented by distribution-based approaches. A clearly established, disease-specific MCID for improvement or decline for MoCA, MDRS-2, and NMSS in PD could not be identified.

## Results

### Search results and study characteristics

**Fig. 1:**
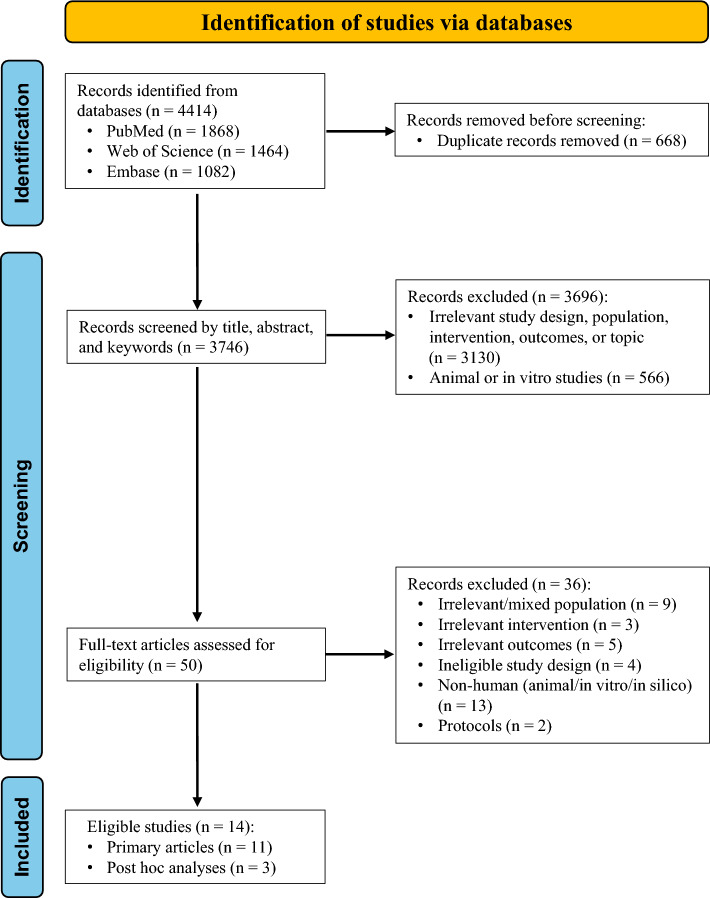
PRISMA flowchart of the study selection process

A total of 4414 records were identified through a database search. After removing duplicates and screening titles and abstracts, 50 full-text articles were assessed for eligibility. Fourteen studies met the inclusion criteria (Fig. 1). Of these, 11 primary articles were included in the quantitative MA (Aviles-Olmos et al. 2013; Gejl et al. 2016; Athauda et al. 2017; Watson et al. 2019; Mullins et al. 2019; Hogg et al. 2022; McGarry et al. 2024; Meissner et al. 2024; Dei Cas et al. 2024; Vijiaratnam et al. 2025; Edison et al. 2025). Three post hoc analyses were considered qualitatively (Aviles-Olmos et al. 2014; Gejl et al. 2017; Athauda et al. 2018).

All of the included trials were RCTs. Most trials were double-blind, except for an open-label MCI trial (Dei Cas et al. 2024) and a single-rater blinded PD proof-of-concept trial (Aviles-Olmos et al. 2013). Detailed study characteristics are summarized in Table 1. Participants’ mean ages ranged from 59.5 to 74.0 years. The percentage of female participants ranged from 16.7% to 69.0%. Where reported, baseline BMI was generally between 25.1 and 30.2 kg/m^2^. The detailed baseline characteristics are summarized in Table S3.

**Table 1:** Study characteristics

Study ID	Trial registration ID	Country	Settings	Phase	Neurodegenerative condition	Diagnostic criteria used	Sample size	Analyzed population	Treatment	Comparator	Study duration (months)
Gejl et al. (2016)	NCT01469351	Denmark	Single center	First-in-human mechanistic RCT	Mild-to-moderate AD	MMSE 18–21; MMSE > 22 required CSF biomarker confirmation	38	Per-protocol	Liraglutide 1.8 mg once daily	Placebo	6
Mullins et al. (2019)	NCT01255163	United States	Single-center	Phase 2	AD spectrum: amnestic MCI and mild AD	CSF Aβ_42_ < 192 pg/mL	27	Modified intention-to-treat	Exenatide 10 μg twice daily	Placebo	18
Watson et al. (2019)	NR	United States	Single-center	Phase 2	Cognitively normal, non-demented individuals at risk for AD	Subjective cognitive complaints, MMSE > 27, no diagnosis of MCI or dementia	41	Per-protocol	Liraglutide 1.8 mg once daily	Placebo	3
Dei Cas et al. (2024)	NCT02847403	Italy	Single-center	Proof-of-concept pilot study	MCI, with or without dysglycemia/prediabetes	Subjective complaint, objective impairment, preserved ADL, no dementia; MMSE 24–27	32	Intention-to-treat	Exenatide 2 mg once weekly	Usual clinical care	8
Edison et al. (2025)	NCT01843075; ISRCTN89711766	United Kingdom	Multicenter	Phase 2b	Mild-to-moderate AD	NINCDS-ADRDA criteria	204	Intention-to-treat + observed cases	Liraglutide 1.2 mg once daily	Placebo	13
Aviles-Olmos et al. (2013)	NCT01174810	United Kingdom	Single-center	Phase 2	Idiopathic PD, moderate stage, levodopa-responsive	Queen Square Brain Bank criteria	44	Modified intention-to-treat	Exenatide 10 μg twice daily + Conventional PD medication	Conventional PD medication	14
Athauda et al. (2017)	NCT01971242	United Kingdom	Single-center	Phase 2	Idiopathic PD, moderate stage	Queen Square Brain Bank criteria	62	Modified intention-to-treat	Exenatide 2 mg once weekly	Placebo	15
Hogg et al. (2022)	NCT02953665	United States	Single-center	Phase 2	Idiopathic PD	UK Brain Bank criteria; disease duration ≥ 2 years or DaTscan consistent with PD	63	Full analysis set using last observation carried forward	Liraglutide 1.2 or 1.8 mg as tolerated once daily	Placebo	13
McGarry et al. (2024)	NCT04154072	United States	Multicenter	Phase 2	Early, untreated idiopathic PD	UK Brain Bank or MDS research criteria, DaTscan confirmation consistent with PD	255	Modified intention-to-treat	NLY01 2.5 mg and NLY01 5.0 mg weekly	Placebo	9
Meissner et al. (2024)	NCT03439943	France	Multicenter	Phase 2	Early PD	UK Brain Bank criteria	156	Modified intention-to-treat	Lixisenatide 20 μg once daily	Placebo	14
Vijiaratnam et al. (2025)	ISRCTN14552789	United Kingdom	Multicenter	Phase 3	PD, moderate stage, receiving dopaminergic treatment	Queen Square Brain Bank criteria; Hoehn and Yahr ≤ 2.5 (ON), MoCA ≥ 21, and PHQ-9 < 16	194	Intention-to-treat	Exenatide 2 mg once weekly	Placebo	24

*AD* Alzheimer’s disease, *Aβ*_*42*_ amyloid-β 42, *ADL* activities of daily living, *CSF* cerebrospinal fluid, *DaTscan* dopamine transporter scan, *MCI* mild cognitive impairment, *MDS* Movement Disorder Society, *MMSE* Mini-Mental State Examination, *MoCA* Montreal Cognitive Assessment, *NINCDS-ADRDA* National Institute of Neurological and Communicative Disorders and Stroke–Alzheimer’s Disease and Related Disorders Association, *NR* not reported, *ON* on-medication state, *PD* Parkinson’s disease, *PHQ-9* Patient Health Questionnaire-9, *RCT* randomized controlled trial, *UK* United Kingdom

### Quality assessment and level of evidence

Of the 11 RCTs, 2 (18.18%) trials (McGarry et al. 2024; Vijiaratnam et al. 2025) were rated as having a low RoB and 8 (72.73%) trials as having some concerns, mainly due to missing outcome data (Gejl et al. 2016; Mullins et al. 2019; Hogg et al. 2022; Edison et al. 2025), deviations from intended interventions (Aviles-Olmos et al. 2013; Meissner et al. 2024; Dei Cas et al. 2024), and baseline imbalances between groups (Aviles-Olmos et al. 2013; Athauda et al. 2017). One trial (9.09%) was rated as having a high RoB, as attrition was substantial and incompletely balanced between groups, with withdrawals frequently related to treatment-associated AEs or tolerability (Figs. S1, S2) (Watson et al. 2019). The certainty of evidence is summarized in Table S4. The GRADE ratings indicated high-certainty evidence for global cognition and verbal fluency. Nine outcomes were graded as having moderate certainty, mainly due to imprecision, and eight outcomes were graded as having low certainty, mainly due to inconsistency and imprecision.

### Primary outcome

#### Global cognition

**Fig. 2:**
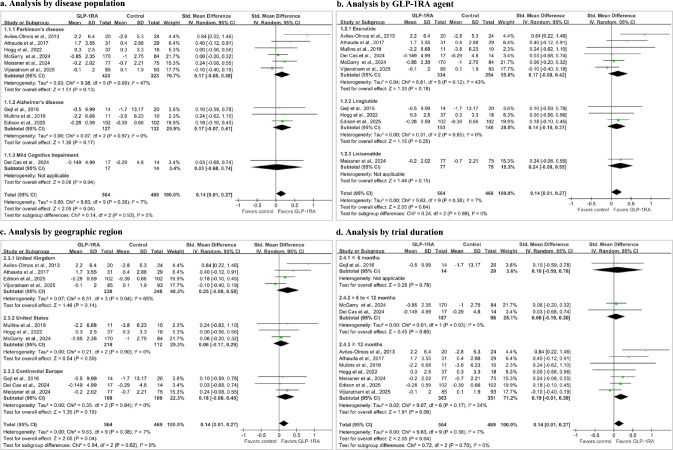
Forest plots of global cognitive outcomes comparing GLP-1RAs versus placebo or standard care. Subgroup analyses are shown by **a** disease population, **b** GLP-1RA agent, **c** geographic region, and **d** trial duration. Effect estimates are presented as SMDs with 95% CIs. Positive SMD values indicate better global cognitive performance with GLP-1RAs than with control. The vertical line at 0 indicates no difference between groups. *CI* confidence interval, *GLP-1RA* glucagon-like peptide-1 receptor agonist, *SMD* standardized mean difference

**Fig. 3:**
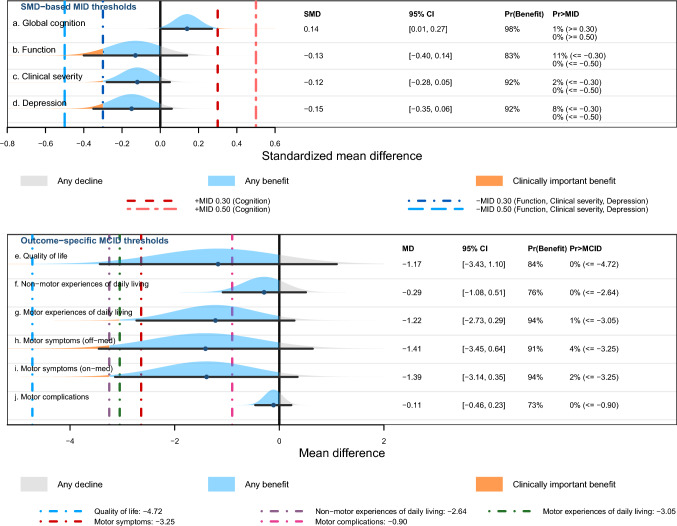
Distribution-based MID and outcome-specific MCID interpretation of pooled efficacy estimates across clinical outcomes. The upper panel shows pooled effects expressed as SMDs for (a) global cognition, (b) function, (c) clinical severity, and (d) depression. These were interpreted against prespecified standardized MID thresholds of 0.30 and 0.50 SD units, applied as + 0.30 and + 0.50 for global cognition and as − 0.30 and − 0.50 for function, clinical severity, and depression according to the direction of improvement. Accordingly, for global cognition, values above 0 favor GLP-1RA treatment, whereas for function, clinical severity, and depression, values below 0 favor GLP-1RA treatment. The lower panel shows pooled effects expressed as MDs for outcome-specific analyses of (e) quality of life, (f) non-motor experiences of daily living, (g) motor experiences of daily living, (h) motor symptoms (off-medication), (i) motor symptoms (on-medication), and (j) motor complications. These were interpreted against published anchor-based MCID thresholds on the original measurement scales. In each row, the density curve represents the distribution of the pooled effect estimate. Shaded regions indicate any decline, any benefit, and clinically important benefit relative to control. Vertical dashed lines indicate the corresponding MID or MCID thresholds. The point estimate and horizontal line represent the pooled effect and 95% CI. The probability columns [Pr(Benefit) and Pr > MID/MCID] represent the likelihood that the true effect is beneficial or exceeds the clinically meaningful threshold, respectively, based on the proportion of the distribution falling beyond those specific lines. *CI* confidence interval, *GLP-1RA* glucagon-like peptide-1 receptor agonist, *MCID* minimal clinically important difference, *MD* mean difference, *MID* minimal important difference, *SMD* standardized mean difference

In the primary MA (10 studies; 1033 participants), GLP-1RAs demonstrated a small but statistically significant benefit in global cognition (SMD 0.14, 95% CI 0.01 to 0.27; p = 0.04; I^2^ = 7%, Fig. 2a). MID-based interpretation suggested a low likelihood of clinically important difference (Pr[SMD ≥ 0.30] = 1% and Pr[SMD ≥ 0.50] = 0%, Fig. 3a). Both funnel plot inspection and Egger’s regression test (z = 0.08, p = 0.94) revealed no evidence of small-study effects (Fig. S3).

Substituting alternative global cognitive measures for trials reporting multiple scales attenuated the pooled effect to non-significance (SMD 0.14, 95% CI − 0.03 to 0.31; I^2^ = 38%, Fig. S4a). However, excluding the MCI trial restored statistical significance (SMD 0.17, 95% CI 0.03 to 0.31; p = 0.02; I^2^ = 12%, Fig. S4b). Leave-one-out analyses demonstrated a generally stable point estimate (SMD range 0.10–0.19, I^2^ range 0–17%, Table S5). The pooled effect remained statistically significant only when omitting 5 trials (Gejl et al. 2016; Hogg et al. 2022; McGarry et al. 2024; Dei Cas et al. 2024; Vijiaratnam et al. 2025). Omitting the remaining studies resulted in the loss of statistical significance (Table S5). Incorporating post-washout cognitive values from the 2 PD trials that reported them (Aviles-Olmos et al. 2013; Athauda et al. 2017) did not alter the statistically significant benefit favoring GLP-1RAs (Fig. S5).

#### Analysis by disease population

Analysis by disease population showed similar effect estimates in PD (6 studies; 743 participants) and AD (3 studies; 259 participants). There was moderate heterogeneity between PD studies (I^2^ = 47%). In contrast, the MCI subgroup (1 study; 31 participants) was highly imprecise. The test for subgroup differences was not significant (p = 0.93, Fig. 2a).

#### Analysis by GLP-1RA agent

Analysis by GLP-1RA agent showed modest numerical variation, with the largest point estimate for lixisenatide (1 study; 152 participants; SMD 0.24, 95% CI − 0.08 to 0.55), followed by exenatide (6 studies; 588 participants) and liraglutide (3 studies; 293 participants). However, all CIs crossed the null, and the test for subgroup differences was not significant (p = 0.89, Fig. 2b).

#### Analysis by geographic region

In the analysis by geographic region, the largest point estimate was in the United Kingdom (4 studies; 486 participants; SMD 0.25, 95% CI − 0.08 to 0.58), followed by Continental Europe (3 studies; 217 participants). The United States (3 studies; 330 participants) had the smallest effect size, and the CI was highly imprecise. Heterogeneity was substantial in the United Kingdom subgroup (I^2^ = 65%) and absent in the other regional subgroups. The test for subgroup differences was not significant (p = 0.62, Fig. 2c).

#### Analysis by trial duration

Analysis by trial duration showed the largest point estimate in trials with ≥ 12 months’ follow-up (7 studies; 714 participants; SMD 0.19, 95% CI − 0.01 to 0.39), compared with > 6 to < 12 months (2 studies; 285 participants) and ≤ 6 months (1 study; 34 participants). The test for subgroup differences was not significant (p = 0.70, Fig. 2d).

#### Instrument-specific analysis

Within the PD population, the pooled estimate for the MDRS-2 (3 studies; 159 participants) non-significantly favored GLP-1RA (MD 1.61, 95% CI − 0.57 to 3.79; I^2^ = 68%). The effect on the MoCA (3 studies; 584 participants) was also imprecise (MD 0.12, 95% CI − 0.29 to 0.52; I^2^ = 17%, Fig. S6).

Within the MCI and mild AD populations, the pooled estimate for MMSE (2 studies; 52 participants) non-significantly favored the control group (MD − 1.19, 95% CI − 4.08 to 1.69; I^2^ = 54%). An MCID-based interpretation suggested a 7% probability of clinically important improvement and a 55% probability of clinically important decline. For ADAS-Cog (2 studies; 52 participants), results non-significantly favored GLP-1RA (MD − 0.55, 95% CI − 3.48 to 2.38; I^2^ = 0%). There was a 17% chance of a clinically important improvement, alongside a 4% risk of a clinically important decline (Fig. S6).

### Secondary outcomes

#### Cognitive subdomains

For verbal fluency, across 4 studies (133 participants), GLP-1RAs significantly worsened verbal fluency (SMD − 0.43, 95% CI − 0.79 to − 0.08; I^2^ = 0%, Fig. S7a). This finding was robust to exclusion of the high-RoB trial (Table S6). In contrast, excluding the trial enrolling prediabetic MCI participants attenuated the effect to non-significance (SMD − 0.37, 95% CI − 0.80 to 0.06; I^2^ = 8%, Table S7). For executive function, verbal learning, and attention, there were no significant differences between treatment groups (Fig. S7b–d). Upon excluding the high-RoB study from these outcomes, the estimate remained imprecise (Table S6). Evidence for visual memory was limited to a single study (26 participants) with an imprecise effect estimate (Fig. S7e).

#### Function

Across 9 studies (999 participants), GLP-1RAs showed no statistically significant difference in function compared with control (SMD − 0.13, 95% CI − 0.40 to 0.14; I^2^ = 73%, Fig. S8a). Estimates differed by disease subgroup; in PD, the pooled estimate favored GLP-1RAs but was heterogeneous and imprecise (SMD − 0.27, 95% CI − 0.62 to 0.08; I^2^ = 79%). In AD, the pooled estimate was centered near the null (SMD − 0.03, 95% CI − 0.29 to 0.23; I^2^ = 0%). In contrast, the single MCI trial showed a large effect in the opposite direction, with a positive SMD indicating significantly better function in the control group (SMD 0.87, 95% CI 0.13 to 1.62). There was evidence of subgroup difference (p = 0.02, Fig. S8a). The probability of achieving a clinically meaningful benefit was low (MID ≤  − 0.30 SD: 11%; MID ≤  − 0.50 SD: 0%, Fig. 3b).

Sensitivity analyses supported the impact of removing the MCI and prediabetic-enrolling trial, yielding a pooled estimate that favored GLP-1RAs; however, it did not reach statistical significance (8 studies; SMD − 0.20, 95% CI − 0.46 to 0.06; I^2^ = 70%, Table S7). Using alternative functional measures when multiple scales were available within a trial produced a similar, non-significant overall estimate (SMD − 0.14, 95% CI − 0.41 to 0.13; I^2^ = 72%, Fig. S9).

#### Clinical severity

Across 6 trials (717 participants), GLP-1RAs were associated with a small non-statistically significant reduction in clinical severity compared with control (SMD − 0.12, 95% CI − 0.28 to 0.05; I^2^ = 10%, Fig. S8b). Subgroup analyses by disease population showed a larger but imprecise effect in PD (SMD − 0.20, 95% CI − 0.44 to 0.04; I^2^ = 31%). In AD, the effect was close to null (SMD − 0.08, 95% CI − 0.34 to 0.18; I^2^ = 0%). The single MCI trial favored control and was imprecise (SMD 0.41, 95% CI − 0.30 to 1.13). There was no evidence of subgroup differences. In the sensitivity analysis that excluded the prediabetic-enrolling trial, the pooled estimate strengthened slightly but remained not statistically significant (SMD − 0.14, 95% CI − 0.30 to 0.01), with heterogeneity reduced to zero (I^2^ = 0%, Table S7). The probability of achieving a clinically meaningful improvement was low (Pr[SMD ≤  − 0.30] = 2% and Pr[SMD ≤  − 0.50] = 0%, Fig. 3c). In the subgroup analysis of outcomes assessed on CDR-SoB in the AD population only, the pooled estimate numerically favored GLP-1RA, but the effect was small and imprecise (MD − 0.18, 95% CI − 0.71 to 0.36; I^2^ = 0%, Fig. S10). The probability of clinically important improvement or decline was 0% (Fig. S10).

#### Depression

Across 5 trials (n = 368), the pooled effect non-significantly favored GLP-1RAs (SMD − 0.15, 95% CI − 0.35 to 0.06; I^2^ = 0%). When stratified by population, the estimate was almost similar in PD trials (SMD − 0.18, 95% CI − 0.42 to 0.07; I^2^ = 15%), while the single MCI trial was highly imprecise (SMD − 0.10, 95% CI − 0.81 to 0.60, Fig. S8c). The probability of achieving a clinically meaningful improvement was low (Pr[SMD ≤  − 0.30] = 8%; Pr[SMD ≤  − 0.50] = 0%, Fig. 3d). In subgroup analysis of outcomes assessed on MADRS in the PD population, the pooled estimate significantly favored GLP-1RA (MD − 2.09, 95% CI − 3.99 to − 0.20; I^2^ = 0%). However, it did not achieve any clinically important improvement (Fig. S10).

#### PD-related outcomes

**Fig. 4:**
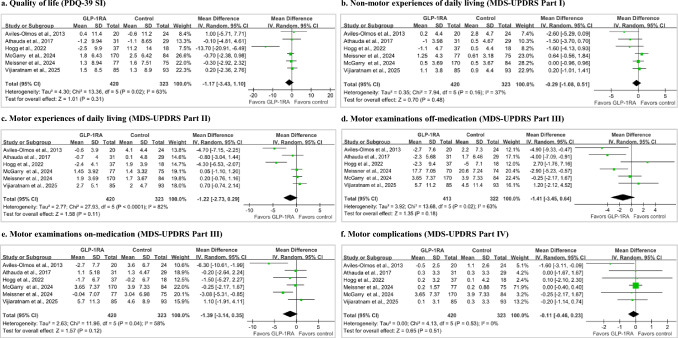
Effects of GLP-1RAs on Parkinson’s disease-related outcomes. Forest plots show the pooled effects with 95% CIs, expressed as mean differences. Outcomes include **a** quality of life (PDQ-39 SI), **b** non-motor experiences of daily living (MDS-UPDRS Part I), **c** motor experiences of daily living (MDS-UPDRS Part II), **d** motor examinations off-medication (MDS-UPDRS Part III), **e** motor examinations on-medication (MDS-UPDRS Part III), and **f** motor complications (MDS-UPDRS Part IV). For all outcomes, effect estimates below 0 indicate improvement with GLP-1RA treatment. The vertical line at 0 indicates no difference between groups. *CI* confidence interval, *GLP-1RA* glucagon-like peptide-1 receptor agonist, *MDS-UPDRS* Movement Disorder Society-sponsored Revision of the Unified Parkinson’s Disease Rating Scale, *PDQ-39 SI* Parkinson’s Disease Questionnaire-39 Summary Index

Across 6 PD trials, the pooled effect demonstrated a non-significant trend favoring GLP-1RAs across all clinical domains. Substantial between-study heterogeneity was observed for QoL (I^2^ = 63%), motor experiences of daily living (I^2^ = 82%), and both off-medication (I^2^ = 63%) and on-medication (I^2^ = 58%) motor examinations. Heterogeneity was low for non-motor experiences of daily living and absent for motor complications outcome (Fig. 4). MCID-based interpretations revealed that the probability of achieving a clinically important improvement was minimal across all domains (ranging from 0% to 4%, Fig. 3e–j), and no outcomes reached the threshold for a clinically important decline (Fig. S11). Across 4 PD trials (547 participants), GLP-1RAs did not significantly improve non-motor symptoms measured using NMSS (MD − 0.54, 95% CI − 4.24 to 3.16; I^2^ = 24%, Fig. S12).

#### Metabolic outcome

Changes in BMI were reported in 3 trials (107 participants). Overall, GLP-1RAs were associated with a significant reduction in BMI (MD − 1.70, 95% CI − 2.44 to − 0.95; I^2^ = 0%, Fig. S13). When the high-RoB trial was excluded, the effect was robust, with only a minimal change in magnitude (Table S6).

#### Levodopa equivalent daily dose

For the levodopa equivalent daily dose (LEDD), the pooled effect of 4 trials (434 participants) showed a small non-significant reduction in LEDD (Fig. S14).

#### Safety and tolerability

**Fig. 5:**
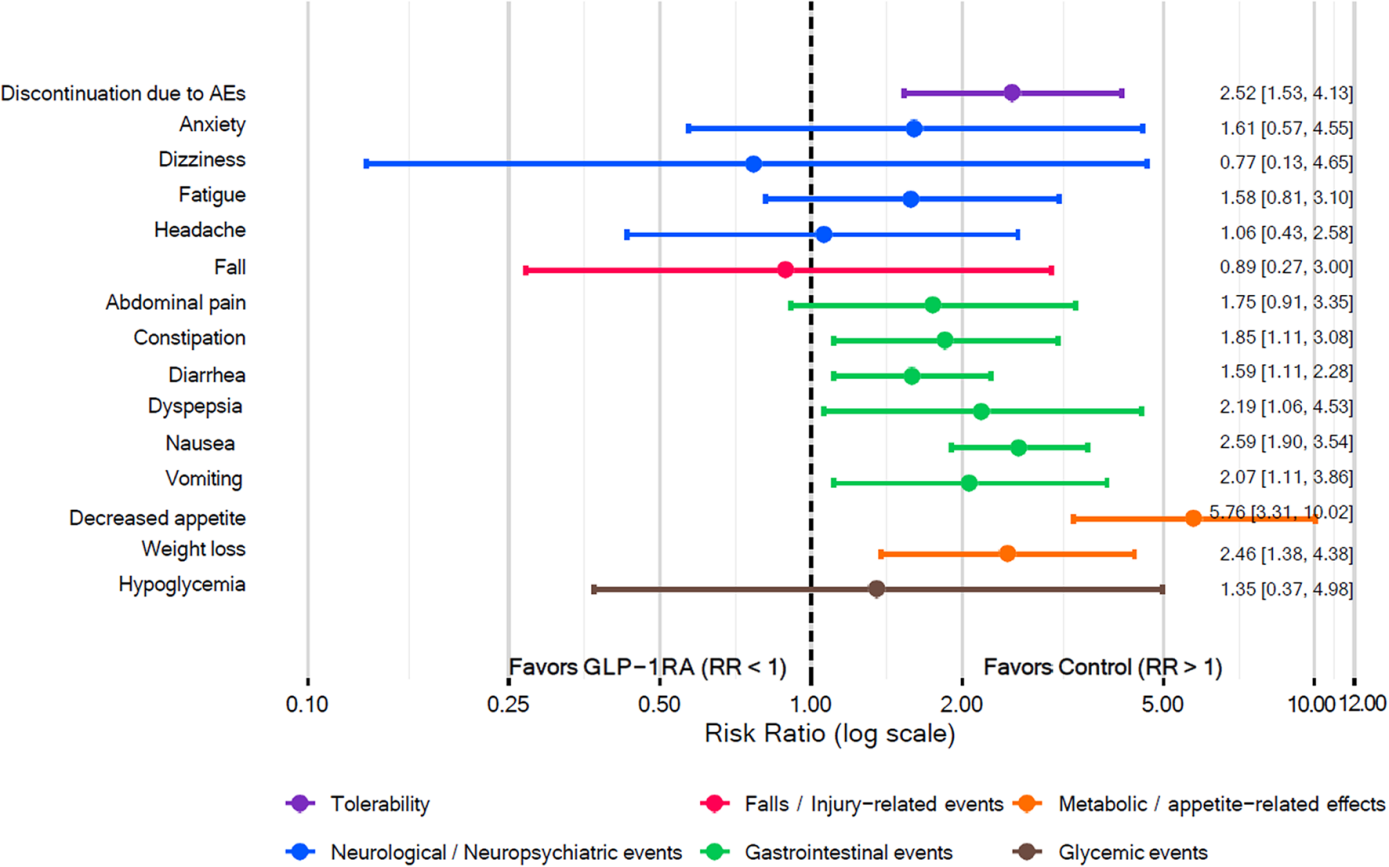
Adverse events associated with GLP-1RAs versus control. Forest plot of pooled RRs with 95% CIs for discontinuation due to adverse events and selected adverse events. The vertical dashed line at RR = 1 indicates no difference between groups. Estimates to the left of this line favor GLP-1RA treatment (lower risk), whereas estimates to the right favor control (higher risk with GLP-1RA treatment). *CI* confidence interval, *GLP-1RA* glucagon-like peptide-1 receptor agonist, *RR* risk ratio

Among the safety outcomes, GLP-1RAs were significantly associated with more gastrointestinal AEs, including constipation, diarrhea, dyspepsia, nausea, and vomiting, decreased appetite and weight loss. In contrast, no statistically significant differences were observed for anxiety, dizziness, fatigue, headache, falls, or hypoglycemia. Overall drug tolerability was lower with GLP-1RAs, with a higher risk of treatment discontinuation due to AEs compared with control (Fig. 5; Figs. S15–S17). As shown in Table 2, these relative effects corresponded to clinically relevant absolute increases in several gastrointestinal and tolerability outcomes, with mostly moderate- to high-certainty evidence.

**Table 2:** Summary of findings for safety and tolerability outcomes of GLP-1 receptor agonists versus control

Outcome	Anticipated absolute effects		Risk ratio (95% CI)	Absolute effect	Participants (studies)	Certainty of evidence (GRADE)	Comments
	Risk with control	Risk with GLP-1RA					
Discontinuation due to AEs	39 Per 1000	97 Per 1000 (59 to 121)	2.52 (1.53 to 4.13)	59 More per 1000 (20 more to 121 more)	1034 (7)	High ⨁⨁⨁⨁	GLP-1RAs increase discontinuation due to adverse events
Headache	41 Per 1000	43 Per 1000 (18 to 105)	1.06 (0.43 to 2.58)	2 More per 1000 (23 fewer to 64 more)	413 (3)	Moderate^a^ ⨁⨁⨁◯	GLP-1RAs probably result in little to no difference in headache, but the estimate is imprecise
Dizziness	114 Per 1000	88 Per 1000 (15 to 531)	0.77 (0.13 to 4.65)	26 Fewer per 1000 (99 fewer to 417 more)	90 (2)	Moderate^a^ ⨁⨁⨁◯	GLP-1RAs probably result in little to no difference in dizziness, but the estimate is imprecise
Fatigue	82 Per 1000	130 Per 1000 (67 to 255)	1.58 (0.81 to 3.10)	48 More per 1000 (16 fewer to 173 more)	751 (4)	Moderate^a^ ⨁⨁⨁◯	GLP-1RAs probably increase fatigue, but the estimate is imprecise
Anxiety	29 Per 1000	47 Per 1000 (17 to 133)	1.61 (0.57 to 4.55)	18 More per 1000 (13 fewer to 104 more)	361 (4)	Moderate^a^ ⨁⨁⨁◯	GLP-1RAs probably increase anxiety, but the estimate is imprecise
Falls	82 Per 1000	73 Per 1000 (22 to 246)	0.89 (0.27 to 3.00)	9 Fewer per 1000 (60 fewer to 164 more)	361 (4)	Moderate^a^ ⨁⨁⨁◯	GLP-1RAs probably result in little to no difference in falls, but the estimate is imprecise
Nausea	159 Per 1000	412 Per 1000 (302 to 562)	2.59 (1.90 to 3.54)	253 More per 1000 (143 more to 404 more)	748 (7)	High ⨁⨁⨁⨁	GLP-1RAs increase nausea
Vomiting	46 Per 1000	95 Per 1000 (51 to 177)	2.07 (1.11 to 3.86)	49 More per 1000 (5 more to 131 more)	677 (5)	High ⨁⨁⨁⨁	GLP-1RAs increase vomiting
Diarrhea	110 Per 1000	174 Per 1000 (122 to 250)	1.59 (1.11 to 2.28)	65 More per 1000 (12 more to 140 more)	748 (7)	High ⨁⨁⨁⨁	GLP-1RAs increase diarrhea
Constipation	136 Per 1000	251 Per 1000 (150 to 417)	1.85 (1.11 to 3.08)	115 More per 1000 (15 more to 282 more)	565 (5)	Moderate^b^ ⨁⨁⨁◯	GLP-1RAs probably increase constipation, although the magnitude of effect varied across studies
Abdominal pain	73 Per 1000	128 Per 1000 (67 to 245)	1.75 (0.91 to 3.35)	55 More per 1000 (7 fewer to 172 more)	325 (4)	Moderate^a^ ⨁⨁⨁◯	GLP-1RAs probably increase abdominal pain, but the estimate is imprecise
Dyspepsia	45 Per 1000	98 Per 1000 (47 to 203)	2.19 (1.06 to 4.53)	53 More per 1000 (3 more to 158 more)	423 (3)	High ⨁⨁⨁⨁	GLP-1RAs increase dyspepsia
Decreased appetite	42 Per 1000	241 Per 1000 (138 to 419)	5.76 (3.31 to 10.02)	199 More per 1000 (97 more to 377 more)	592 (6)	High ⨁⨁⨁⨁	GLP-1RAs increase the risk of decreased appetite
Weight loss	144 Per 1000	353 Per 1000 (198 to 629)	2.46 (1.38 to 4.38)	210 More per 1000 (55 more to 486 more)	740 (7)	Moderate^b^ ⨁⨁⨁◯	GLP-1RAs probably increase weight loss, although the magnitude of effect varied across studies
Hypoglycemia	27 Per 1000	36 Per 1000 (10 to 132)	1.35 (0.37 to 4.98)	9 More per 1000 (17 fewer to 106 more)	246 (3)	Moderate^a^ ⨁⨁⨁◯	GLP-1RAs probably result in little to no difference in hypoglycemia, but the estimate is imprecise

The risk with GLP-1RA (and its 95% CI) is based on the assumed risk in the control group and the relative effect of the intervention (and its 95% CI)

*AE* adverse event, *CI* confidence interval, *GLP-1RA* glucagon-like peptide-1 receptor agonist, *GRADE* Grading of Recommendations, Assessment, Development and Evaluation

^a^Imprecision (downgraded 1 level): downgraded when the95% CI crossed the line of no effect and included both appreciable benefit or harm

^b^Inconsistency(downgraded 1 level): downgraded when there was unexplained between-study heterogeneity

### Risk–benefit profile of GLP-1RAs across efficacy and safety outcomes

In terms of absolute effects per 1000 patients, GLP-1RA therapy was associated with pronounced increases in gastrointestinal and tolerability outcomes, most notably nausea (+ 253), weight loss (+ 210), and decreased appetite (+ 199). Drug tolerability corresponded to an absolute increase of 59 discontinuations per 1000 patients. Fewer patients treated with GLP-1RAs experienced dizziness (− 26) and falls (− 9). For efficacy outcomes, absolute effects were estimated using prespecified MCID thresholds, representing the number of patients per 1000 who achieved a clinically important improvement. The absolute efficacy gains corresponded to 116 more patients per 1000 for depression, 70 more for PD motor experiences of daily living, and 54 more for PD motor examination on-medication with GLP-1RA treatment compared with control. Conversely, for cognition measured by the MMSE, 124 fewer patients reached the clinically important difference compared with control (Fig. S18).

### Meta-regression analysis

Meta-regression using a random-effects model (REML with Knapp–Hartung adjustment) showed no evidence that treatment duration influenced the global cognition effect size, with an estimated slope of β = 0.00 SMD per week (SE = 0.00; p = 0.64), corresponding to an approximate change of − 0.02 SMD per 10 weeks. After accounting for duration, residual heterogeneity was low (τ^2^ = 0.01; I^2^ = 18.9%). Similarly, disease group did not significantly moderate the effect, with PD studies showing a non-significant difference compared with AD-spectrum studies (β = 0.16; SE = 0.22; p = 0.49) and residual heterogeneity remained moderate (τ^2^ = 0.03; I^2^ = 41.5%). CIs for heterogeneity parameters were wide (duration model τ^2^ 95% CI 0.00 to 0.51; I^2^ 95% CI 0.0% to 91.4%; disease model τ^2^ 95% CI 0.00 to 0.42; I^2^ 95% CI 0.0% to 89.9%, Fig. S19).

### Narrative synthesis of study findings

#### Parkinson’s disease

For PD, the findings were inconsistent across trials. Early proof-of-concept studies on exenatide suggested modest improvements in off-medication motor severity, with signals persisting through extended follow-ups, alongside improvements in cognition, while dopaminergic imaging changes were minimal (Aviles-Olmos et al. 2013, 2014). Subsequent double-blind evidence again favored exenatide for motor outcomes off-medication and reported a signal consistent with a slower decline in striatal DAT binding (Athauda et al. 2017). Post hoc analyses of non-motor outcomes were exploratory and many signals were not sustained after withdrawals (Athauda et al. 2018). In contrast, the large phase 3 trial found no meaningful benefits across motor, cognitive, functional, QoL, or dopaminergic imaging outcomes (Vijiaratnam et al. 2025). Other agents showed domain-specific patterns; liraglutide improved function and patient-reported QoL without clear effects on global cognition or motor severity (Hogg et al. 2022). Lixisenatide improved the motor endpoint, with benefits persisting after washout; however, non-motor and cognitive outcomes were similar between the groups (Meissner et al. 2024). NLY01 did not improve any primary or secondary outcomes, with tolerability concerns at higher doses (McGarry et al. 2024). Further details are listed in Table S8.

#### Alzheimer’s disease and mild cognitive impairment

In AD and MCI, evidence for GLP-1RAs remains mixed, with signals of biological activity but limited translation into clinical efficacy. In the non-diabetic MCI population, weekly exenatide did not improve cognition, function, or behavioral measures compared with the control group, and exploratory analyses suggested possible sex-related heterogeneity, including potential worsening among female participants (Dei Cas et al. 2024). In the trial enrolling MCI and mild AD, exenatide was terminated early and underpowered, with no discernible benefits on cognition, daily functioning, magnetic resonance spectroscopy metabolites, structural magnetic resonance imaging (MRI) measures, or CSF biomarkers; a minor biochemical signal in plasma extracellular vesicle-derived Aβ_42_ was not accompanied by clinical improvement (Mullins et al. 2019). Liraglutide trials have demonstrated target engagement or neurobiological signals more consistently than clinical benefits. A 26-week liraglutide trial in mild-to-moderate AD did not reduce Aβ deposition or improve cognition relative to placebo, but FDG-PET suggested preserved cerebral glucose metabolism in AD-vulnerable regions (Gejl et al. 2016). Over a 52-week trial, liraglutide did not meet the primary FDG-PET metabolic change endpoint, although exploratory analyses suggested modest benefits for executive function-specific measures and MRI signals (Edison et al. 2025). In an early intervention mechanistic trial, liraglutide administration over 12 weeks in middle-aged adults at risk of dementia did not improve performance in neuropsychological testing, but resting-state functional magnetic resonance imaging (fMRI) showed enhanced hippocampal-default mode network connectivity (Watson et al. 2019). Further details are listed in Table S8.

## Discussion

This SR and MA included 14 RCTs with 1260 participants, restricted to non-diabetic cohorts diagnosed with AD, MCI, or PD. GLP-1RAs were associated with a small but statistically significant improvement in global cognition, supported by high-certainty evidence. However, MID-based exploratory analysis suggested that the probability of this effect translating into a clinically meaningful cognitive benefit was trivial. For secondary outcomes, including function, clinical severity, depression, and PD motor and non-motor symptoms, pooled estimates generally favored GLP-1RAs but were not statistically significant. However, in a PD subgroup analysis, GLP-1RAs significantly improved depression symptoms, although this improvement did not clearly exceed the MCID threshold. From a safety and tolerability perspective, GLP-1RAs were associated with higher rates of gastrointestinal AEs, decreased appetite, weight loss, and lower tolerability. Conversely, fewer falls and dizziness events were observed in the GLP-1RA group than in controls.

Preclinical studies have suggested that GLP-1RAs may improve cognitive function (Katsenos et al. 2022). Despite encouraging findings from preclinical and observational studies, evidence from clinical trials remains inconsistent regarding the cognitive benefits of GLP-1RAs (Wah Lam et al. 2026). Across the individual RCTs included in our analysis, cognitive effects were inconsistent and mostly non-significant, except for one study in which the cognitive benefit persisted following a 2-month washout period (Aviles-Olmos et al. 2013). Notably, when pooled across all the included trials, GLP-1RAs showed a small statistically significant cognitive benefit. This is consistent with a MA limited to PD populations that reported a statistically significant cognitive benefit versus placebo, although that review did not include the latest PD trials (Albuquerque et al. 2025). By contrast, a recent MA restricted to AD and MCI trials found no significant treatment effect (O’Mara et al. 2026). Given that cognition was not the primary outcome in most included studies, except one trial (Dei Cas et al. 2024), individual RCTs were often not designed or powered to detect modest cognitive effects. Therefore, statistical significance may only emerge after pooling, particularly in the presence of small sample sizes (Button et al. 2013).

In the domain-specific analyses, we observed no clear benefits for executive function, verbal learning and memory, visual memory, attention, or working memory, whereas verbal fluency favored the control group. These findings, however, should be interpreted cautiously given the limited evidence base for each domain and the heterogeneity in cognitive tests and populations. In addition, short-term treatment tolerability may influence performance on some cognitive tests, complicating interpretation of isolated domain-level signals.

In AD models, GLP-1RAs have been associated with reduced Aβ pathology and tau phosphorylation (Katsenos et al. 2022). However, biomarker effects in the included trials were inconsistent. One trial found no reduction in amyloid burden on amyloid PET (Gejl et al. 2016). In contrast, two trials suggested positive metabolic and mechanistic signals, including the attenuation of cerebral glucose hypometabolism (measured via FDG-PET) and increased blood–brain barrier glucose transport capacity (Gejl et al. 2016, 2017). Another trial reported a significant group-by-visit effect for extracellular vesicle Aβ_42_, although this occurred without corresponding improvements in core CSF biomarkers (Mullins et al. 2019). Taken together, these findings suggest possible CNS target engagement, but they do not provide consistent evidence that GLP-1RAs modify core AD pathological processes in a way that clearly translates into clinical benefit.

In a 12-week placebo-controlled trial, liraglutide increased resting-state functional connectivity between the hippocampus and several cortical clusters overlapping with the default mode network. This pattern is consistent with the hypothesis that liraglutide may mitigate adverse effects of glucose dysregulation on brain network function. Since these networks are crucial for memory and cognition, such changes may reflect a biologically plausible neural response to treatment. This potential makes liraglutide particularly relevant for individuals at elevated AD risk due to modifiable factors such as insulin resistance or non-modifiable factors such as family history. However, no parallel improvements were detected in objective cognitive performance or subjective complaints over the same period. Therefore, these fMRI findings are best interpreted as target engagement signals rather than evidence of clinical efficacy (Watson et al. 2019). Studies with larger samples and longer follow-up periods are required to determine whether such network-level modulations translate into detectable cognitive benefits in a relatively cognitively unimpaired population.

In rodent models, central GLP-1 receptor activation has been associated with reduced anxiety- and depression-like behaviors. In humans, a previous MA of 5 RCTs reported that GLP-1RAs significantly improved depressive symptoms in a mixed diabetic and non-diabetic population (Chen et al. 2024). In our analysis, the pooled estimate favored GLP-1RAs but did not reach statistical significance. Subgroup analysis, however, showed significant improvement in depressive symptoms in PD. Nevertheless, MCID-based analysis indicates that the observed improvement still falls below the threshold for clinically important benefit. This suggests that although a treatment signal may be present, its magnitude is likely modest and may not be large enough to produce a noticeable or decision-relevant benefit for most individual patients. Therefore, current evidence points to a modest exploratory antidepressant effect rather than a robust clinical benefit. The mechanisms underlying this potential effect also remain uncertain (Chen et al. 2024).

In our analysis, GLP-1RAs did not significantly improve functional abilities or global clinical outcomes. Evidence from individual trials remains inconsistent. For example, in AD, the ELAD trial reported no significant benefit on functional status or global severity. Similarly, a recent SR concluded that the available clinical evidence for GLP-1RAs in AD is still limited and does not demonstrate a consistent efficacy signal on clinical endpoints (Liang et al. 2024). According to 2018 FDA draft guidance, treatment efficacy in mild-to-moderate dementia is typically expected to include concurrent improvement in cognition, function, and clinician-based global impression (Hamilton et al. 2025). Within that framework, the current evidence does not indicate that GLP-1RAs meet a multi-domain threshold suggestive of clinically established benefit. For PD, some trials have reported improvements in daily activities, suggesting possible symptomatic benefits in selected domains (Aviles-Olmos et al. 2013; Hogg et al. 2022). This mixed pattern may reflect limited statistical power, short follow-up periods, or true heterogeneity in treatment responsiveness across populations and outcome domains.

Across PD outcomes, pooled effects generally favored GLP-1RAs but did not reach statistical significance. Early exenatide trials reported clinically meaningful improvements in motor severity off-medication that persisted after washout (Aviles-Olmos et al. 2013; Athauda et al. 2017). However, these findings were not replicated by the large 96-week phase 3 trial (Vijiaratnam et al. 2025). Findings for non-motor symptoms and QoL outcomes were similarly inconsistent. While a few trials reported domain-specific improvements, most trials—particularly the more recent ones—found no significant advantages. Even where pooled estimates trended positively, the probability of an individual patient achieving an MCID across these domains remained very low. Overall, current evidence does not demonstrate a convincing disease-modifying signal in PD. GLP-1RAs may exert metabolic, neuroinflammatory, or neuroprotective effects within the CNS, but these remain speculative and insufficient to yield consistent or clinically meaningful benefit against established neurodegeneration (Athauda et al. 2026).

Dopaminergic imaging has provided exploratory yet inconclusive evidence of disease modification. One study reported a DaT-SPECT signal suggestive of a slower decline in the striatal dopamine transporter binding (Athauda et al. 2017). However, these observations should be interpreted with caution as apparent persistence may reflect symptomatic effects or bias. Moreover, DaT imaging has limited sensitivity and specificity as a progression biomarker (Zarkali et al. 2024). Importantly, larger, more recent trials have not confirmed the benefits of dopaminergic imaging (McGarry et al. 2024; Vijiaratnam et al. 2025).

In pooled analyses of LEDD, GLP-1RAs did not significantly reduce dopaminergic medication requirements compared with control. Meaningful disease modification would ideally slow escalation of dopaminergic medication over time. Therefore, the lack of a consistent LEDD-sparing effect provides limited support for a robust symptomatic dopaminergic benefit or a clear disease-modifying signal within the study period. However, LEDD is an indirect outcome influenced by prescribing practices, symptom targets, and tolerability. Therefore, small effects, if present, may require larger samples and longer follow-up periods to be detected (Julien et al. 2021).

Safety findings suggest that GLP-1RA use is often limited by gastrointestinal intolerance and catabolic effects, such as reduced appetite and weight loss. Pooled evidence showed a higher risk of discontinuation due to AEs. Weight loss induced by GLP-1RAs should be interpreted in a patient-specific context. In insulin-resistant cohorts, BMI reduction may reflect beneficial metabolic effects, consistent with improved insulin sensitivity (Watson et al. 2019). Conversely, in AD and PD cohorts, additional weight loss was not necessarily advantageous (Mullins et al. 2019; Hogg et al. 2022). Although frailty and sarcopenia were not analyzed, weight loss in these populations may overlap with these vulnerability states or disease-related decline, reflecting prognosis rather than therapeutic benefit (Ibrahim et al. 2025). A recent commentary highlighted the potential of GLP-1RAs in worsening nutritional deficits in vulnerable populations. Evidence also links disrupted energy homeostasis to accelerated neurodegeneration, suggesting that even modest weight loss can have unfavorable cognitive consequences (Ibrahim et al. 2025). The associated AEs may have important clinical consequences by reducing oral intake and worsening hydration, thereby limiting the feasibility of sustained use in routine practice (Ismaiel et al. 2025). Overall, the current evidence suggests that treatment burden may outweigh the magnitude of demonstrated clinical benefit. Accordingly, the broader clinical utility of GLP-1RAs remains uncertain, and use is better regarded as investigational.

This study has several strengths. We focused specifically on non-diabetic populations, reducing confounding from glycemic control and allowing a clearer interpretation of the potential neuroprotective effects. Our methods were transparent and protocolized, and we evaluated a broad set of clinically relevant endpoints. The inclusion of both efficacy and safety endpoints enabled a comprehensive assessment of the overall benefit–risk profile of GLP-1RAs. Importantly, we complemented conventional MA estimates using the MID/MCID framework to facilitate clinical interpretation. This allows us to shift the focus from purely statistical significance to clinical relevance, ensuring that pooled results reflect changes that are actually meaningful to patients rather than just mathematically detectable. In addition, we conducted extensive sensitivity analyses to confirm the robustness of our results and subgroup analyses to explore efficacy differences across key study and participant characteristics.

Nevertheless, our study has several limitations. Owing to strict eligibility criteria, only 14 trials were included. Follow-up durations varied across trials. However, meta-regression found no duration–effect relationship, suggesting that differences in follow-up alone are unlikely to explain the inconsistent results. Pooling AD, PD, and MCI in a single primary analysis may have oversimplified important differences in pathophysiology, cognitive trajectories, and the validity and sensitivity of the instruments used across these conditions. Accordingly, the pooled estimate should be interpreted as an overall cross-condition summary rather than as evidence of a uniform treatment effect across populations. Disease-specific MID evidence for AD and PD was limited; therefore, we prespecified distribution-based thresholds informed by broader MID literature from other populations. Another limitation of this approach is that distribution-based methods are purely statistical. While they effectively capture mathematical variability within a sample, they do not inherently confirm whether a calculated change is clinically meaningful to the patient (Ousmen et al. 2018). Therefore, the distribution-based interpretations were regarded as exploratory rather than definitive. Finally, the included trials were conducted in Western populations, which may limit generalizability to other ethnic groups and regions.

Future trials should prioritize earlier-stage or prevention settings and enroll larger cohorts with longer follow-up. Incorporating biologically enriched populations, such as insulin-resistant or inflammatory phenotypes, and selecting endpoints aligned to the proposed mechanism with clinically meaningful anchors will be essential. A greater emphasis on safety and tolerability should be considered. Structured support strategies, including gradual dose titration, preemptive management of AEs, nutritional oversight, and caregiver engagement, may be essential to optimize retention and reduce attenuation of potential treatment effects. Collectively, these design features will be important for determining whether any observed benefit is symptomatic or disease-modifying.

In parallel, several key areas require further investigation to strengthen the application of MCID in neurodegenerative trials. First, there remains a notable lack of anchor-based validation for several cognitive measures, such as the MDRS-2 and MoCA. Furthermore, even among well-established clinical measures, such as CDR-SoB and ADAS-Cog, existing anchor-based literature predominantly focuses on thresholds for disease deterioration rather than improvement. A critical future direction is to establish MCID thresholds for clinical improvement (Hamilton et al. 2025). Regulatory agencies increasingly support the use of anchor-based methods to determine the clinical meaningfulness of individual-level change. Robust and disease-specific MCID estimates should be leveraged within regulatory submission packages to better contextualize treatment effects.

In conclusion, this study suggests that GLP-1RAs may confer a modest cognitive benefit; however, the effect appears to have limited clinical relevance. Evidence across other clinical outcomes and biomarkers was inconsistent and did not support any clear symptomatic or disease-modifying effect. Any potential benefit must also be weighed against weight loss and frequent AEs. Definitive large-scale trials are needed to clarify the benefit–risk profile and identify subgroups most likely to benefit.

## Supplementary Information

Below is the link to the electronic supplementary material.

Supplementary file1 (DOCX 2850 KB)

## Data Availability

All data generated or analyzed during this study are included in this published article and its supplementary information files.
