# Supplementary material for: Evaluating the clinical effects of GLP-1 receptor agonists for Alzheimer's and Parkinson's diseases using minimal clinically important difference: systematic review and meta-analysis
Source: Arch Pharm Res. 2026 May 7;49(5):691–712. doi: 10.1007/s12272-026-01615-y (PMC13222307; doi:10.1007/s12272-026-01615-y)
Supplement: Supplementary file 1 — Supplementary file1 (DOCX 2850 KB) [file 12272_2026_1615_MOESM1_ESM.docx]

**Evaluating the Clinical Effects of GLP-1 Receptor Agonists for Alzheimer's and Parkinson's Diseases Using Minimal Clinically Important Difference: Systematic Review and Meta-analysis**

Yomna Elghanam^1, a^, EunYoung Kim^1, 2, 3, b, *^

^1^ *Data Science, Evidence-Based and Clinical Research Laboratory, Department of Health, Social, and Clinical Pharmacy, College of Pharmacy, Chung-Ang University, Seoul, 06974, Republic of Korea*

^2^ *The Graduate School for Pharmaceutical Industry Management, College of Pharmacy, Chung-Ang University, Seoul, 06974, Republic of Korea*

^3^ *Department of Pharmaceutical Regulatory Science, College of Pharmacy, Chung-Ang University, Seoul, 06974, Republic of Korea*

^*^ *Corresponding author. Evidence-Based and Clinical Research Laboratory, Department of Health, Social, and Clinical Pharmacy, College of Pharmacy, Chung-Ang University, 84 Heukseok-ro, Dongjak-gu, Seoul, 06974, Republic of Korea.* E-mail address: [eykimjcb777@cau.ac.kr](mailto:eykimjcb777@cau.ac.kr)

^a^ ORCiD <https://orcid.org/0009-0007-9467-1313>

^b^ ORCiD <https://orcid.org/0000-0003-3525-8805>

**Running heading:** GLP-1RAs in Non-Diabetic Neurodegeneration

[PROSPERO registration number] CRD420261277032

# **Table S1.** PRISMA checklist:

| **Section and Topic** | **Item #** | **Checklist item** | **Location where item is reported** |
| --- | --- | --- | --- |
| **TITLE** | | |  |
| Title | 1 | Identify the report as a systematic review. | 1 |
| **ABSTRACT** | | |  |
| Abstract | 2 | See the PRISMA 2020 for Abstracts checklist. | 2 |
| **INTRODUCTION** | | |  |
| Rationale | 3 | Describe the rationale for the review in the context of existing knowledge. | 6 |
| Objectives | 4 | Provide an explicit statement of the objective(s) or question(s) the review addresses. | 6 |
| **METHODS** | | |  |
| Eligibility criteria | 5 | Specify the inclusion and exclusion criteria for the review and how studies were grouped for the syntheses. | 8 |
| Information sources | 6 | Specify all databases, registers, websites, organisations, reference lists and other sources searched or consulted to identify studies. Specify the date when each source was last searched or consulted. | 8-9, Table S2 |
| Search strategy | 7 | Present the full search strategies for all databases, registers and websites, including any filters and limits used. | Table S2 |
| Selection process | 8 | Specify the methods used to decide whether a study met the inclusion criteria of the review, including how many reviewers screened each record and each report retrieved, whether they worked independently, and if applicable, details of automation tools used in the process. | 10 |
| Data collection process | 9 | Specify the methods used to collect data from reports, including how many reviewers collected data from each report, whether they worked independently, any processes for obtaining or confirming data from study investigators, and if applicable, details of automation tools used in the process. | 10 |
| Data items | 10a | List and define all outcomes for which data were sought. Specify whether all results that were compatible with each outcome domain in each study were sought (e.g. for all measures, time points, analyses), and if not, the methods used to decide which results to collect. | 10-14 |
|  | 10b | List and define all other variables for which data were sought (e.g. participant and intervention characteristics, funding sources). Describe any assumptions made about any missing or unclear information. | 10, 15 |
| Study risk of bias assessment | 11 | Specify the methods used to assess risk of bias in the included studies, including details of the tool(s) used, how many reviewers assessed each study and whether they worked independently, and if applicable, details of automation tools used in the process. | 13 |
| Effect measures | 12 | Specify for each outcome the effect measure(s) (e.g. risk ratio, mean difference) used in the synthesis or presentation of results. | 14 |
| Synthesis methods | 13a | Describe the processes used to decide which studies were eligible for each synthesis (e.g. tabulating the study intervention characteristics and comparing against the planned groups for each synthesis (item #5)). | 9-16 |
|  | 13b | Describe any methods required to prepare the data for presentation or synthesis, such as handling of missing summary statistics, or data conversions. | 14 |
|  | 13c | Describe any methods used to tabulate or visually display results of individual studies and syntheses. | 14 |
|  | 13d | Describe any methods used to synthesize results and provide a rationale for the choice(s). If meta-analysis was performed, describe the model(s), method(s) to identify the presence and extent of statistical heterogeneity, and software package(s) used. | 14 |
|  | 13e | Describe any methods used to explore possible causes of heterogeneity among study results (e.g. subgroup analysis, meta-regression). | 14 |
|  | 13f | Describe any sensitivity analyses conducted to assess robustness of the synthesized results. | 14 |
| Reporting bias assessment | 14 | Describe any methods used to assess risk of bias due to missing results in a synthesis (arising from reporting biases). | 14 |
| Certainty assessment | 15 | Describe any methods used to assess certainty (or confidence) in the body of evidence for an outcome. | 13 |
| **RESULTS** | | |  |
| Study selection | 16a | Describe the results of the search and selection process, from the number of records identified in the search to the number of studies included in the review, ideally using a flow diagram. | 18 |
|  | 16b | Cite studies that might appear to meet the inclusion criteria, but which were excluded, and explain why they were excluded. | NA |
| Study characteristics | 17 | Cite each included study and present its characteristics. | 18, Table 1 |
| Risk of bias in studies | 18 | Present assessments of risk of bias for each included study. | 18, Fig. S1-S2 |
| Results of individual studies | 19 | For all outcomes, present, for each study: (a) summary statistics for each group (where appropriate) and (b) an effect estimate and its precision (e.g. confidence/credible interval), ideally using structured tables or plots. | 20-30 |
| Results of syntheses | 20a | For each synthesis, briefly summarise the characteristics and risk of bias among contributing studies. | Table S4 |
|  | 20b | Present results of all statistical syntheses conducted. If meta-analysis was done, present for each the summary estimate and its precision (e.g. confidence/credible interval) and measures of statistical heterogeneity. If comparing groups, describe the direction of the effect. | 20-30 |
|  | 20c | Present results of all investigations of possible causes of heterogeneity among study results. | 20-30 |
|  | 20d | Present results of all sensitivity analyses conducted to assess the robustness of the synthesized results. | 20-30 |
| Reporting biases | 21 | Present assessments of risk of bias due to missing results (arising from reporting biases) for each synthesis assessed. | 20, Fig. S3 |
| Certainty of evidence | 22 | Present assessments of certainty (or confidence) in the body of evidence for each outcome assessed. | 18, Table 2, Table S4 |
| **DISCUSSION** | | |  |
| Discussion | 23a | Provide a general interpretation of the results in the context of other evidence. | 34 |
|  | 23b | Discuss any limitations of the evidence included in the review. | 41 |
|  | 23c | Discuss any limitations of the review processes used. | 41 |
|  | 23d | Discuss implications of the results for practice, policy, and future research. | 34-43 |
| **OTHER INFORMATION** | | |  |
| Registration and protocol | 24a | Provide registration information for the review, including register name and registration number, or state that the review was not registered. | 8 |
|  | 24b | Indicate where the review protocol can be accessed, or state that a protocol was not prepared. | 8 |
|  | 24c | Describe and explain any amendments to information provided at registration or in the protocol. | NA |
| Support | 25 | Describe sources of financial or non-financial support for the review, and the role of the funders or sponsors in the review. | 44 |
| Competing interests | 26 | Declare any competing interests of review authors. | 44 |
| Availability of data, code and other materials | 27 | Report which of the following are publicly available and where they can be found: template data collection forms; data extracted from included studies; data used for all analyses; analytic code; any other materials used in the review. | 44 |

# **Table S2:** Search strategy

| **Item** | **Search term** | **Number** | **Date** |
| --- | --- | --- | --- |
| **PubMed** | | |  |
| #1 | "Glucagon-Like Peptide 1 Receptor Agonists"[MeSH Terms] OR "glucagon like peptide 1 receptor agonist*"[Title/Abstract] OR "glp 1 receptor agonist*"[Title/Abstract] OR "incretin*"[Title/Abstract] OR "semaglutide"[Title/Abstract] OR "liraglutide"[Title/Abstract] OR "exenatide"[Title/Abstract] OR "dulaglutide"[Title/Abstract] OR "albiglutide"[Title/Abstract] OR "lixisenatide"[Title/Abstract] OR "efpeglenatide"[Title/Abstract] OR "benaglutide"[Title/Abstract] OR "beinaglutide"[Title/Abstract] OR "taspoglutide"[Title/Abstract] | 22,537 | 2025/11/12 |
| #2 | "Dementia"[MeSH Terms] OR "Alzheimer Disease"[MeSH Terms] OR "parkinson disease/complications"[MeSH Terms] OR "Dementia"[Title/Abstract] OR "alzheimer*"[Title/Abstract] OR "vascular dementia"[Title/Abstract] OR "parkinson* dementia"[Title/Abstract] OR "Lewy"[Title/Abstract] OR "MCI"[Title/Abstract] OR "cognitive decline"[Title/Abstract] OR "global cognition"[Title/Abstract] OR "MMSE"[Title/Abstract] OR "Mini-Mental State"[Title/Abstract] OR "ADAS-Cog"[Title/Abstract] OR "MoCA"[Title/Abstract] OR "WAIS"[Title/Abstract] OR "incident dementia"[Title/Abstract] OR "MCI conversion"[Title/Abstract] OR "cerebrospinal fluid"[Title/Abstract] OR "CSF"[Title/Abstract] OR "plasma"[Title/Abstract] OR "serum"[Title/Abstract] OR "extracellular vesicle*"[Title/Abstract] OR "exosome*"[Title/Abstract] OR "neuron derived extracellular vesicle*"[Title/Abstract] OR "amyloid beta"[Title/Abstract] OR "Abeta"[Title/Abstract] OR "tau"[Title/Abstract] OR "phospho-tau"[Title/Abstract] OR "p-tau"[Title/Abstract] OR "insulin receptor substrate-1"[Title/Abstract] OR "IRS-1"[Title/Abstract] OR "cerebral glucose metabolism"[Title/Abstract] OR "FDG-PET"[Title/Abstract] OR "brain glucose metabolism"[Title/Abstract] | 2,925,486 | 2025/11/12 |
| #3 | "humans"[MeSH Terms] NOT ("Review"[Publication Type] OR "Systematic Review"[Publication Type]) | 20,165,828 | 2025/11/12 |
| #4 | #1 AND #2 AND #3 | 1,868 | 2025/11/12 |
| **Embase** | | |  |
| #1 | 'glucagon like peptide 1 receptor agonist'/exp OR ('glucagon like':ti,ab,kw AND 'peptide 1':ti,ab,kw AND receptor:ti,ab,kw AND agonist*:ti,ab,kw) OR 'glp-1 receptor agonist*':ti,ab,kw OR incretin*:ti,ab,kw OR semaglutide:ti,ab,kw OR liraglutide:ti,ab,kw OR exenatide:ti,ab,kw OR dulaglutide:ti,ab,kw OR albiglutide:ti,ab,kw OR lixisenatide:ti,ab,kw OR efpeglenatide:ti,ab,kw OR benaglutide:ti,ab,kw OR beinaglutide:ti,ab,kw OR taspoglutide:ti,ab,kw | 74,316 | 2025/11/12 |
| #2 | 'dementia'/exp OR 'alzheimer disease'/exp OR 'vascular dementia'/exp OR ('parkinson disease'/exp AND (dementia:ti,ab,kw OR complication:ti,ab,kw)) OR dementia:ti,ab,kw OR alzheimer*:ti,ab,kw OR 'vascular dementia':ti,ab,kw OR 'parkinson* dementia':ti,ab,kw OR lewy:ti,ab,kw OR 'mild cognitive impairment'/exp OR 'mild cognitive impairment':ti,ab,kw OR mci:ti,ab,kw OR 'cognitive decline':ti,ab,kw OR 'global cognition':ti,ab,kw OR mmse:ti,ab,kw OR 'mini-mental state':ti,ab,kw OR 'adas-cog':ti,ab,kw OR moca:ti,ab,kw OR wais:ti,ab,kw OR 'incident dementia':ti,ab,kw OR 'mci conversion':ti,ab,kw OR 'cerebrospinal fluid':ti,ab,kw OR csf:ti,ab,kw OR plasma:ti,ab,kw OR serum:ti,ab,kw OR 'extracellular vesicle*':ti,ab,kw OR exosome*:ti,ab,kw OR 'neuron-derived extracellular vesicle*':ti,ab,kw OR 'amyloid beta':ti,ab,kw OR aβ:ti,ab,kw OR tau:ti,ab,kw OR 'phospho-tau':ti,ab,kw OR 'p-tau':ti,ab,kw OR 'cerebral glucose metabolism':ti,ab,kw OR 'fdg pet':ti,ab,kw OR 'brain glucose metabolism':ti,ab,kw OR 'insulin receptor substrate-1':ti,ab,kw OR 'irs 1':ti,ab,kw | 4,090,363 | 2025/11/12 |
| #3 | #1 AND #2 AND [embase]/lim NOT ([embase]/lim AND [medline]/lim) AND 'human'/de NOT ('animal'/exp NOT 'human'/exp) NOT ('conference abstract'/it OR 'conference review'/it OR 'conference paper'/it OR 'review'/it OR 'systematic review'/it) | 1,082 | 2025/11/12 |
| **Web of Science** | | |  |
| #1 | TS=(("glucagon-like peptide-1" NEAR/3 receptor* NEAR/3 agonist*) OR "GLP-1 receptor agonist*" OR semaglutide OR liraglutide OR exenatide OR dulaglutide OR albiglutide OR lixisenatide OR efpeglenatide OR benaglutide OR beinaglutide OR taspoglutide)  AND  TS=(dementia OR Alzheimer* OR "vascular dementia" OR "Parkinson* dementia" OR lewy OR "mild cognitive impairment" OR MCI OR "MCI conversion" OR "incident dementia" OR "cognitive decline" OR "global cognition" OR MMSE OR "Mini-Mental State" OR "ADAS-Cog" OR MoCA OR WAIS OR "cerebrospinal fluid" OR CSF OR plasma OR serum OR "extracellular vesicle*" OR exosome* OR "neuron-derived extracellular vesicle*" OR "amyloid beta" OR Abeta OR tau OR "phospho-tau" OR "p-tau" OR "cerebral glucose metabolism" OR "brain glucose metabolism" OR FDG-PET OR "insulin receptor substrate-1" OR IRS-1)  AND  DT=(Article)  NOT  TS=(mouse OR mice OR murine OR rat OR rats OR rodent*) | 1,464 | 2025/11/12 |

# **Table S3.** Baseline characteristics

| **Study ID** | **Intervention** | | | | | | | **Comparator** | | | | | | |
| --- | --- | --- | --- | --- | --- | --- | --- | --- | --- | --- | --- | --- | --- | --- |
|  | **Drug name** | **Sample size** | **Age, years mean ± SD** | **Sex female (%)** | **Baseline disease severity** | **BMI/ body weight (kg)** | **Other key baseline features** | **Comparator** | **Sample size** | **Age,**  **years mean ± SD** | **Sex female (%)** | **Baseline disease severity** | **BMI /body weight (kg)** | **Other key baseline features** |
| Gejl et al.,2016 | Liraglutide | 14 | 63.1 ± 4.9 | 57% | Mild–moderate AD. **WMS-IV total score** 27.1 (range 5–44; SEM 3.4) | Body weight 74.1 ± 7.9; BMI 25.1 ± 3.7 | Background AD treatments permitted; cholinesterase inhibitors could be continued if initiated before enrolment and stable | Placebo | 20 | 66.6 ± 8.0 | 25% | **WMS-IV total score** 27.2 (range 0–57; SEM 3.8) | Body weight 77.8 ± 13.4; BMI 25.1 ± 3.13 | NR |
| Mullins etal., 2019 | Exenatide | 11 | 71.7 ± 6.9 | 36% | AD spectrum. **MMSE** 25.5 ± 4.2; **CDR–SoB** 2.9 ± 1.7 | BMI 27.1 ± 3.4 kg/m² | Fasting glucose: 89.6 ± 7.4 mg/dL, Fasting insulin: 8.1 ± 4.6 μIU/mL | Placebo | 10 | 74.0 ± 6.4 | 60% | **MMSE** 26.0 ± 3.5; **CDR-SoB** 2.5 ± 1.5 | BMI 26.9 ± 5.9 kg/m² | Fasting glucose 93.8 ± 8.4 mg/dL, Fasting insulin 83 ± 4.8 μIU/mL |
| Watson etal., 2019 | Liraglutide | 25 | 60.9 ± 5.8 | 56% | Cognitively normal, non-demented individuals. **MMSE** >27; subjective cognitive complaints without objective impairment; no CDR staging | BMI 30.22 ± 6.04 kg/m² | Mixed insulin-sensitive and insulin-resistant participants; approximately half had a first-degree family history of dementia. Major depression and other significant psychiatric or neurological disorders were excluded. Fasting plasma glucose 95.55 (8.44) mg/dL,  OGTT 120-min glucose 115.39 (32.46) mg/dL,  Fasting plasma insulin 25.95 (42.73) μIU/mL | Placebo | 16 | 59.6 ± 5.7 | 69% | Cognitively normal; **MMSE** >27 | BMI 29.24 ± 6.43 kg/m² | Fasting plasma glucose 98.69 (9.59) mg/Dl;  OGTT 120-min glucose 112.73 (23.21) mg/dL,  Fasting plasma insulin 9.04 (7.53) μIU/mL |
| Dei Cas et al., 2024 | Exenatide | 17 | 74.0 ± 4.0 | 50% | MCI (amnestic). **ADAS-Cog11** 14.05 ± 5.5; **MMSE** 25.9 ± 1.3; **CDR** 0.26 ± 0.26; non-demented; AD aetiology not biomarker-confirmed | Body weight 70.5 ± 10.5 kg; BMI 27.0 ± 3.6 kg/m² | Dysglycemia present in 29.4% (FPG 100–125 mg/dL and/or HbA1c 39–46 mmol/mol).  Common comorbidities included hypertension (~47%), dyslipidemia (50%), gastrointestinal disorders (50%), and mild neuropsychiatric conditions (31%), balanced between groups. Concomitant medications were mainly cardiovascular (lipid-lowering 50%, antihypertensives 31%, antithrombotics 31%, antidepressants/anxiolytics 44%). All women were post-menopausal | Usual clinical care | 15 | 72.0 ± 6.0 | 53% | **ADAS-Cog11** 12.16 ± 5.44; **MMSE** 26.1 ± 1.8; **CDR** 0.30 ± 0.32 | Body weight 75.8 ± 11.4 kg; BMI 27.6 ± 3.3 kg/m² | Very similar profile to exenatide group: dysglycemia in 40%; comparable rates of hypertension, dyslipidemia, gastrointestinal and mild neuropsychiatric comorbidities, and similar concomitant cardiovascular medications. |
| Edison et al., 2025 | Liraglutide | 102 | 70.6 ± 8.4 | 41% | Mild–moderate AD. **MMSE** 23.7 ± 3.5; **CDR–SoB** 3.7 ± 1.9; **ADAS-Cog13** 31.6 ± 10.3 | NR | Non-diabetic participants with mild–moderate AD; baseline functional status ADCS-ADL 66.5 ± 9.5 and neuropsychiatric symptom burden NPI 10.2 ± 12.5 | Placebo | 102 | 72.5 ± 7.0 | 38% | **MMSE** 23.4 ± 3.6; **CDR-SoB** 3.6 ± 1.8; **ADAS-Cog13** 31.9 ± 9.3 | NR | Non-diabetic participants with mild–moderate AD; functional status ADCS-ADL 66.1 ± 9.7; neuropsychiatric symptoms NPI 7.5 ± 9.0 |
| Aviles-Olmos et al., 2013 | Exenatide + standard of care | 20 | 61.4 ± 6.0 | 25% | PD. Hoehn & Yahr (on) **stage 2** (n=14) and **stage 2.5** (n=6) | NR | Disease duration 9.6 ± 3.4 years | Standard of care | 24 | 59.4 ± 8.4 | 16.70% | Hoehn & Yahr (on): **stage 2** (n=16), **stage 2.5** (n=8) | NR | Disease duration 11.0 ± 5.9 years |
| Athauda et al., 2017 | Exenatide | 31 | 61.6 ± 8.2 | 29% | PD. Hoehn & Yahr (on) **stage 1.0–2.0 in** 94% and **stage 2.5 in** 6%; **MDS-UPDRS Part III (off)** mean 32.8; no dementia at baseline (MDRS ≥120) | BMI ≥18.5 kg/m² | Disease duration ≈ 6.4 ± 3.3 years | Placebo | 29 | 57.8 ± 8.0 | 24% | Hoehn & Yahr (on) stage **1·0–2·0:** all participants; MDS-UPDRS Part III (**off) mean** 27.1 ± 10.3; no dementia at baseline (MDRS ≥120) | BMI ≥18.5 kg/m² | Disease duration ≈ 6.4 ± 3.3 years |
| Hogg etal., 2022 | Liraglutide | 37 | 63.5 ± 9.8 | 32.40% | PD. Symptom duration 4.7 ± 3.1 years; **MDS-UPDRS Part III (off)** 26.1 ± 9.6; **MDRS**-2 140.1 ± 3.2; **NMSS** 32.9 ± 26.8; **LED** 564 ± 327 mg | BMI 28.0 ± 4.3 kg/m² | Predominantly Caucasian population; Prediabetes (HbA1c ≥5.7%) in 40.5% (15/37) | Placebo | 18 | 64.2 ± 6.4 | 27.80% | MDS-UPDRS **Part III (off**) 28.8 ± 10.7; **MDRS-2** 140.7 ± 2.5; **NMSS** 31.1 ± 25.0; **LED** 640 ± 360 mg; symptom duration 4.8 ± 3.3 years | BMI 27.9 ± 4.3 kg/m² | Prediabetes (HbA1c ≥5.7%) in 22.2% (4/18); similar PD duration between groups; slightly lower baseline non-motor symptom severity compared with liraglutide group |
| McGarry, 2024 | NLY01 | NLY01 2.5 mg: 85; NLY01 5.0 mg: 85 | NLY01 2.5 mg: 62.1 ± 9.0; NLY01 5.0 mg: 60.6 ± 10.0 | NLY01 2.5 mg: 29%; NLY01 5.0 mg: 36% | **PD. NLY01 2.5** mg: **Hoehn & Yahr stage** 1 (26%), 1.5 (4%), 2 (67%), 2.5 (4%); **MDS-UPDRS Part III** 22.7 ± 8.1. NLY01 5.0 mg: **Hoehn & Yahr stage 1** (18%), 1.5 (8%), 2 (71%), 2.5 (4%); **MDS-UPDRS** Part III 22.0 ± 8.2 | NLY01 2.5 mg: BMI 26.42 ± 4.14 kg/m²; NLY01 5.0 mg: BMI 25.81 ± 4.58 kg/m² | Very early, untreated PD. Disease duration: 370.9 ± 378.8 days (NLY01 2.5 mg) and 351.6 ± 341.7 days (NLY01 5 mg). Preserved cognition at baseline (MoCA ≥24) | Placebo | 84 | 61.8 ± 8.1 | 38% | MDS-UPDRS **Part I** 4.7 ± 4.2; **Part II** 4.9 ± 3.6; **Part III** 22.3 ± 9.1; **sum of Parts II+III** 27.2 ± 10.3 | BMI 26.03 ± 4.66 kg/m²; body weight 77.8 ± 16.2 kg | Early PD: MoCA ≥24 at screening; Hoehn & Yahr ≤2.5; largely drug-naïve (≤28 days lifetime PD therapy; none within 14 days before baseline) |
| Meissner etal., 2024 | Lixisenatide | 78 | 59.5 ± 8.1 | 44% | PD. **MDS-UPDRS Part III** 14.8 ± 7.3 | BMI 25.6 ± 3.9 kg/m² | Preserved cognition (MoCA 27.8 ± 1.4); short disease duration with mean time since diagnosis of 1.4 years in both groups | Placebo | 78 | 59.9 ± 8.4 | 38% | **MDS-UPDRS Part III** 15.5 ± 7.8 | BMI 25.8 ± 4.2 kg/m² | Preserved cognition at baseline (MoCA 28.1 ± 1.4) |
| Vijiaratnam etal., 2025 | Exenatide | 92 | 61.0 ± 9.1 | 29% | PD. **Hoehn & Yahr (on) ≤2.0** in 86% and stage 2.5 in 14% | BMI median 25.8 kg/m² (IQR 23.5–28.6); body weight 79.6 ± 14.7 kg | Preserved cognition (MoCA ~28), low depressive symptoms (PHQ-9), and NMSS and quality-of-life scores (PDQ-39) typical for moderate PD; participants were able to self-administer weekly injections | Placebo | 97 | 60.4 ± 9.3 | 29% | Hoehn & Yahr (on): **≤2.0** in 85%, **stage 2.5** in 15% | BMI median 25.2 kg/m² (IQR 23.1–28.0); body weight 78.4 ± 13.6 kg | Similar to treatment arm |

AD: Alzheimer’s disease; ADAS-Cog11: Alzheimer’s Disease Assessment Scale–Cognitive Subscale (11-item); ADAS-Cog13: Alzheimer’s Disease Assessment Scale–Cognitive Subscale (13-item); ADCS-ADL: Alzheimer’s Disease Cooperative Study–Activities of Daily Living; BMI: body mass index; CDR: Clinical Dementia Rating; ChEIs: cholinesterase inhibitors; FPG: fasting plasma glucose; HbA1c: glycated hemoglobin (hemoglobin A1c); IQR: interquartile range; LED: levodopa equivalent dose; MCI: mild cognitive impairment; MDRS-2: Mattis Dementia Rating Scale–2; MDS-UPDRS: Movement Disorder Society–Unified Parkinson’s Disease Rating Scale; MMSE: Mini-Mental State Examination; MoCA: Montreal Cognitive Assessment; NMSS: Non-Motor Symptoms Scale; NPI: Neuropsychiatric Inventory; NR: not reported; OGTT: oral glucose tolerance test; PD: Parkinson’s disease; on: “on” medication state; PDQ-39: Parkinson’s Disease Questionnaire-39; PHQ-9: Patient Health Questionnaire-9; SD: standard deviation; SEM: standard error of the mean; WMS-IV: Wechsler Memory Scale–Fourth Edition.

# **Table S4.** GRADE assessment

| **Outcome** | **No. of studies** | **No.of participants (studies)** | **Risk of bias** | **Inconsistency** | **Indirectness** | **Imprecision** | **Other considerations** | **Effect SMD (95%CI)** | **Quality of evidence** |
| --- | --- | --- | --- | --- | --- | --- | --- | --- | --- |
| **Primary outcomes** | | | | | | | | | |
| Cognition | 10 | 1033 | Not serious | Not serious | Not serious | Not serious | Not serious | SMD 0.14 [0.01, 0.27] | ⨁⨁⨁⨁ High |
| Cognition on MMSE | 2 | 52 | Not serious | Serious | Not serious | Serious | Not serious | MD-1.19 [-4.08, 1.69] | ⨁⨁◯◯ Low ^b, d^ |
| Cognition on ADAS-Cog | 2 | 52 | Not serious | Not serious | Not serious | Serious | Not serious | MD -0.55 [-3.48, 2.38] | ⨁⨁⨁◯ Moderate ^d^ |
| Cognition on MDRS-2 | 3 | 159 | Not serious | Serious | Not serious | Serious | Not serious | MD 1.61 [-0.57, 3.79] | ⨁⨁◯◯ Low ^b, d^ |
| Cognition on MoCA | 3 | 584 | Not serious | Not serious | Not serious | Serious | Not serious | MD 0.12 [-0.29, 0.52] | ⨁⨁⨁◯ Moderate ^d^ |
| **Secondary outcomes** | | | | | | | | | |
| Verbal fluency | 4 | 133 | Not serious | Not serious | Not serious | Not serious | Not serious | SMD -0.43 [-0.79, -0.08] | ⨁⨁⨁⨁ High |
| Executive function | 2 | 81 | Not serious | Serious | Not serious | Serious | Not serious | SMD -0.04 [-0.71, 0.64] | ⨁⨁◯◯ Low ^b, d^ |
| Verbal learning & memory | 2 | 60 | Not serious | Not serious | Not serious | Serious | Not serious | 0.30 [-0.21, 0.82] | ⨁⨁⨁◯ Moderate ^d^ |
| Visual memory | 1 | 26 | Not serious | Not serious | Not serious | Serious | Not serious | SMD -0.58 [-1.37, 0.22] | ⨁⨁⨁◯ Moderate ^d^ |
| Attention / working memory | 2 | 47 | Not serious | Not serious | Not serious | Serious | Not serious | SMD 0.42 [-0.16, 1.00] | ⨁⨁⨁◯ Moderate ^d^ |
| Function | 9 | 999 | Not serious | Serious | Not serious | Serious | Not serious | SMD -0.13 [-0.40, 0.14] | ⨁⨁◯◯ Low ^b, d^ |
| Clinical severity | 6 | 717 | Not serious | Not serious | Not serious | Serious | Not serious | SMD -0.12 [-0.28, 0.05] | ⨁⨁⨁◯ Moderate ^d^ |
| Depression | 5 | 368 | Not serious | Not serious | Not serious | Serious | Not serious | SMD -0.15 [-0.35, 0.06] | ⨁⨁⨁◯ Moderate ^d^ |
| PD non-motor symptom burden | 6 | 743 | Not serious | Not serious | Not serious | Serious | Not serious | MD -0.29 [-1.08, 0.51] | ⨁⨁⨁◯ Moderate ^d^ |
| Quality of life | 6 | 743 | Not serious | Serious | Not serious | Serious | Not serious | MD -1.17 [-3.43, 1.10] | ⨁⨁◯◯ Low ^b, d^ |
| PD experiences of daily living | 6 | 743 | Not serious | Serious | Not serious | Serious | Not serious | MD -1.22 [-2.73, 0.29] | ⨁⨁◯◯ Low ^b, d^ |
| PD motor symptoms (off-medication) | 6 | 735 | Not serious | Serious | Not serious | Serious | Not serious | MD -1.41 [-3.45, 0.64] | ⨁⨁◯◯ Low ^b, d^ |
| PD motor symptoms (on-medication) | 6 | 743 | Not serious | Serious | Not serious | Serious | Not serious | MD -1.39 [-3.14, 0.35] | ⨁⨁◯◯ Low ^b, d^ |
| PD motor complications | 6 | 743 | Not serious | Not serious | Not serious | Serious | Not serious | MD -0.11 [-0.46, 0.23] | ⨁⨁⨁◯ Moderate ^d^ |

a. Risk of bias (downgraded 1 level): Downgraded when the contributing evidence showed important methodological limitations, including high risk of bias or common concerns in a key RoB 2 domain.

b. Inconsistency (downgraded 1 level): Downgraded when there was unexplained between-study heterogeneity.

c. Indirectness (downgraded 1 level): Downgraded when the available evidence was not directly applicable to the review question because of differences in the study populations, interventions, comparators, or outcome measures.

d. Imprecision (downgraded 1 level): Downgraded when the 95% CI crossed the line of no effect (SMD = 0) and included both benefit and no effect (or potential harm).

ADAS-Cog, Alzheimer’s disease assessment scale – cognitive; MDRS-2, CI, confidence interval; GLP-1RA, glucagon-like peptide-1 receptor agonist; GRADE, Grading of Recommendations, Assessment, Development and Evaluation; MMSE, Mini-mental state examination; MoCA, Montreal cognitive assessment PD, Parkinson’s disease; RoB 2, Revised Cochrane risk-of-bias tool for randomized trials; SMD, standardized mean difference.

# **Table S5.** Leave-one-out sensitivity analysis for the global cognition outcome

| **Omitted study** | **n** | **Pooled SMD [95% CI]** | **I² (%)** | **Interpretation** |
| --- | --- | --- | --- | --- |
| Aviles-Olmos et al., 2013 | 9 | 0.10 [-0.02, 0.23],  (p = 0.11) | 0% | Slight attenuation of the pooled effect with loss of statistical significance; overall direction unchanged and heterogeneity reduced |
| Athauda et al., 2017 | 9 | 0.12 [-0.02, 0.26],  (p = 0.08) | 7% | Minor attenuation of the pooled effect with loss of statistical significance; heterogeneity similar to the primary analysis |
| Hogg et al., 2022 | 9 | **0.15 [0.01, 0.30],**  **(p = 0.04)** | 15% | Pooled effect slightly strengthened and remained statistically significant; heterogeneity increased modestly |
| McGarry et al., 2024 | 9 | **0.16 [0.01, 0.32],**  **(p = 0.04)** | 13% | Pooled effect increased slightly and remained statistically significant; heterogeneity modestly increased |
| Meissner et al., 2024 | 9 | 0.13 [-0.03, 0.28],  (p = 0.11) | 13% | Effect estimate attenuated with loss of statistical significance; heterogeneity increased modestly |
| Vijiaratnam et al., 2025 | 9 | **0.19 [0.05, 0.32],**  **(p = 0.008)** | 0% | Pooled effect strengthened and remained statistically significant; heterogeneity reduced to zero |
| Gejl et al., 2016 | 9 | **0.14 [-0.00, 0.29],**  **(p = 0.05)** | 17% | Effect estimate unchanged in magnitude but statistical significance was borderline; heterogeneity increased |
| Mullins et al., 2019 | 9 | 0.14 [0.00, 0.28],  (p = 0.06) | 16% | Pooled effect stable but marginal loss of statistical significance; heterogeneity increased |
| Edison et al., 2025 | 9 | 0.14 [-0.02, 0.30],  (p = 0.09) | 16% | Effect size stable with loss of statistical significance; heterogeneity modestly increased |
| Dei Cas et al., 2024 | 9 | **0.15 [0.00, 0.29],**  **(p = 0.05)** | 16% | Pooled effect slightly strengthened but confidence interval included the null; heterogeneity increased modestly |

n, number of studies remaining after omission; SMD, standardized mean difference; CI, confidence interval; I², heterogeneity statistic. **Note:** Original pooled SMD 95%CI: 0.14 [0.01, 0.27]; I² (%): 7%.

# **Table S6.** Sensitivity analysis excluding studies at high risk of bias (RoB)

| **Outcome** | **Primary analysis** | | **Sensitivity analysis** | |
| --- | --- | --- | --- | --- |
|  |  |  | **Excluding studies at high RoB** | |
|  | **n** | **Effect size [95% CI], I^2^** | **n** | **Effect size [95% CI], I^2^** |
| **Verbal fluency** | 4 | SMD -0.43 [-0.79, -0.08], 0% | 3 | SMD -0.52 [-0.92, -0.12], 0% |
| **Executive function** | 2 | SMD -0.04 [-0.71, 0.64], 53% | 1 | SMD 0.26 [-0.31, 0.82] |
| **Attention /working memory** | 2 | SMD 0.42 [-0.16, 1.00], 0% | 1 | SMD 0.46 [-0.41, 1.33] |
| **Verbal learning & memory** | 2 | SMD 0.30 [-0.21, 0.82], 0% | 1 | SMD 0.41 [-0.28, 1.10] |
| **BMI** | 3 | MD -1.70 [-2.44, -0.95], 0% | 2 | MD -1.73 [-2.49, -0.96], 0% |

n, number of studies; SMD, standardized mean difference; MD: mean difference; CI, confidence interval; I², heterogeneity statistic.

# **Table S7.** Sensitivity analysis excluding trials enrolling prediabetic participants

| **Outcome** | **Primary analysis** | | **Excluding the prediabetic trial (Dei Cas et al., 2024)** | |
| --- | --- | --- | --- | --- |
|  | **n** | **SMD [95% CI], I^2^** | **n** | **SMD [95% CI], I^2^** |
| **Global cognition** | 10 | 0.14 [0.01, 0.27], 7% | 9 | **0.15 [0.00, 0.29], 16%** |
| **Verbal fluency** | 4 | -0.45 [-0.79, -0.10], 0% | 3 | -0.37 [-0.80, 0.06], 8% |
| **Function** | 9 | -0.13 [-0.40, 0.14], 73% | 8 | -0.20 [-0.46, 0.06], 70% |
| **Clinical severity** | 6 | -0.12 [-0.28, 0.05], 10% | 5 | **-0.14 [-0.30, 0.01], 0%** |
| **Depression** | 5 | -0.15 [-0.35, 0.06], 0% | 4 | -0.18 [-0.42, 0.07], 16% |

n, number of studies; SMD, standardized mean difference; CI, confidence interval; I², heterogeneity statistic.

# **Table S8.** Results summary

| **Study ID** | **Primary endpoint** | **Cognitive outcomes** | **Other outcomes** | **Neurodegeneration biomarkers** | **Main Findings** | **Conclusions** |
| --- | --- | --- | --- | --- | --- | --- |
| Aviles-Olmos et al., 2013 | Change from baseline in MDS-UPDRS Part III in the off-medication state | MDRS-2 | MDS-UPDRS Parts I, II, and IV, MADRS, PDQ-39 Summary Index, and timed motor tests | [123I]FP-CIT SPECT (DaTscan) | **Tolerability:** Exenatide generally was well tolerated; expected AEs including weight loss (mean ~−3.2 kg vs −0.8 kg controls over 12 months) and GI symptoms.  **Motor:**  Blinded MDS-UPDRS Part III (off) favored exenatide; between-group differences ~4.9 points at 12 months and ~4.4 points at 14 months (both significant). Including open-label rigidity scores increased differences to ~7 points, and 7.2 points at 14 months.  **Global PD burden:**  MDS-UPDRS Parts I/II/IV favored exenatide; combined Parts I–IV advantage ~13.8 at 12 months and ~12.3 at 14 months.  **Cognition:**  MDRS-2 favored exenatide; at 14 months advantage 6.3 (95% CI 2.7–9.9; p=.001).  **Mood/QoL:**  No significant differences in MADRS or PDQ-39.  **DaTscan substudy:**  Minimal overall change; atypical small improvements in a few exenatide cases. | This proof-of-concept single-blind study suggests exenatide is tolerable in moderate PD and that motor and cognitive differences may emerge and persist beyond treatment, potentially consistent with biological (disease-modifying) activity.  Due to small size, single-blind and open-label features, and lack of placebo injections, placebo/expectation and long-duration symptomatic effects cannot be excluded; results justify larger double-blind placebo-controlled GLP-1RA trials in PD and raise interest for testing in neurodegenerative dementias |
| Aviles-Olmos et al., 2014 post hoc analysis | Change from baseline in MDS-UPDRS Part III in the off-medication state | MDRS-2 | MDS-UPDRS Parts I, II, and IV, MADRS, PDQ-39 Summary Index, and timed motor tests | NR | Benefits in previously treated participants persisted 12 months after stopping exenatide.  **Motor:**  At 24 months, exenatide exposure associated with MDS-UPDRS Part III (off, blinded) treatment difference of 5.6 points (95% CI 2.2–9.0; p=.002); including rigidity (open-label) gave ~8.0 point difference (95% CI 3.8–12.2; p<.001).  **Cognition:**  At 24 months, MDRS-2 treatment difference 5.3 points (95% CI 1.4–9.3; p=.006). No correlations were found between age at PD onset and MDRS-2 at baseline nor between change in MDRS-2 at 12, 14 or 24 months  **Other:**  At 24 months, MDS-UPDRS Parts I–II worsened less (significant).  They observed small, non-significant treatment differences favoring the intervention group on several non-motor measures:  **Depression:**  Showed improvement in the intervention group 1.9 points versus worsening in controls 1.5 points but did not reach statistical significance.  **Non motor symptoms questionnaires:**  Similar non-significant patterns were seen for NMS Quest and sleep quality (SCOPA Sleep night-time and overall).  **Quality of life:**  There was no between-group difference in the PDQ-39 summary index at follow-up, and baseline imbalance in PDQ-39 subdomains was noted as a factor that could influence apparent changes.  **Metabolically:**  Treatment-associated weight loss was largely reversible after stopping treatment, with no between-group differences in BMI change at follow-up; both groups showed similar mean weight loss relative to baseline with a difference of 0.1 points, and oral glucose tolerance testing indicated no difference in glucose tolerance between groups. | Persistent motor and cognitive differences after treatment cessation cannot be definitively attributed to neuroprotection given open-label design, baseline imbalances, and possible expectation effects. Nonetheless, the magnitude and durability of signals support larger, double-blind, placebo-controlled GLP-1RA trials in PD to clarify symptomatic versus disease-modifying effects |
| Athauda et al., 2017 | Change from baseline to Week 60 in MDS-UPDRS Part III in the practically defined off-medication state | MDRS-2 total score and subdomains, assessed at Weeks 48 and 60 | MADRS, NMSS, MDS-UPDRS Parts I–II, PDQ  -39, EQ-5D (index and VAS), Hauser diaries, and timed motor tests | Striatal dopaminergic function: DaTscan SPECT | **Motor:**  At Week 60, MDS-UPDRS Part III (off) favored exenatide: exenatide improved ~1.0 point vs placebo worsened ~2.1 points; adjusted between-group difference −3.5 (95% CI −6.7 to −0.3; p=.0318). Benefit was evident at Week 48 (difference −4.3; p=.0026) and persisted after 12-week washout.  **Secondary:**  No significant differences in MDRS/DRS global cognition, overall non-motor burden, UDysRS, or PDQ-39 summary index, or EuroQol Five Dimensions Questionnaire, nor in results on timed motor tests or Hauser diaries.  **LEDD:**  No significant difference was noted in total LEDD at 60 weeks between the groups, the mean increase in LEDD was 19.6 mg higher in the exenatide than in the placebo group.  **Biomarker:**  DaTscan suggested slower decline in striatal DAT signal with exenatide (significant).  **AEs:**  The frequency of adverse effects did not differ significantly between groups. Weight change occurred in both groups but was more common in the exenatide than in the placebo group. | Weekly exenatide produced a sustained advantage in off-state motor severity versus placebo over 60 weeks in moderate PD. Whether this represents a disease-modifying effect or a durable symptomatic benefit remains uncertain; findings support larger and longer-term GLP-1RA trials in PD |
| Athauda et al., 2018 (post hoc analysis | Change from baseline in individual non-motor symptom domains and cognitive domains | NMSS attention/memory domain, MDS-UPDRS Part I Item 1.1 (cognitive impairment), PDQ-39 cognition domain, MDRS-2 total and subdomains; proportion with MCI defined as MDRS-2 <137 | PDQ-39 Summary Index and 8 domains (mobility, ADL, emotional well-being, stigma, social support, cognition, communication, bodily discomfort); EQ-5D (index and VAS) | None  newly analysed | **Post hoc non-motor/QoL:**  At Week 48, exenatide vs placebo showed greater improvement in mood-related measures (NMSS mood/apathy domain ~−3.3 (95% CI –6.2, –0.4); p~.026;  **MDS-UPDRS Part I mood** items Q1.3+Q1.4 ~−0.3 (95%CI –0.6, –0.1); p~.034; MADRS trend ~−1.7 (95%CI –3.6, 0.2); p~.071).  **Quality of life:**  Improved PDQ-39 emotional well-being (~−5.7; (95%CI –11.3, –0.1) p~.047).  **Logistic models** suggested reduced odds of depression/apathy (OR ~0.2–0.25; borderline p~.056–.07).  **Durability:** Mood/apathy signals were not clearly sustained at Week 60 (12 weeks after stopping exenatide).  **Other domains**: No clear effects across most other non-motor domains or broader cognitive scales. | Post hoc analyses suggest exenatide may improve mood/apathy-related outcomes and the PDQ-39 emotional well-being domain at 48 weeks, potentially independent of motor effects. However, results are exploratory (no multiplicity adjustment), many signals were not sustained after withdrawal, and effects were not consistent across non-motor domains.  **Conclusions** are hypothesis-generating and intended to guide outcome selection/powering in future trials |
| Hogg etal., 2022 | Change from baseline to Weeks 28 and 54 in: MDS-UPDRS Part III (off), NMSS, and MDRS-2 | MDRS-2 total score and NMSS attention/memory subdomain | MDS-UPDRS Part II, PDQ-39 total score and subdomains, MDS-UPDRS Global score and Parts I, III, IV | None collected | **Motor:**  **MDS-UPDRS Part III (off medication):** at 54 weeks, scores improved in both groups (liraglutide −2.6 vs placebo −5.0 points), with no significant between-group difference (p =.32).  **Cognition:**  MDRS-2: changes did not significantly differ between groups (week 54 change +0.6 vs +0.3, p =.84).  **Other (non-motor / function / QoL, anxiety):**  **NMSS:** at 54 weeks, NMSS improved in liraglutide (−6.6) and worsened in placebo (+6.5), giving a 13.1-point adjusted mean difference (p <.05).  **Subdomains:** all 9 favored liraglutide; attention/memory subdomain also significant (p <.05).  **MDS-UPDRS Part II:** improved in liraglutide (−2.2) vs worsened in placebo (+1.9), 3.1 adjusted mean difference (p =.001).  **PDQ-39:** improved in liraglutide (−2.5) vs worsened in placebo (+11.2), 13.7 adjusted mean difference (p <.001).  **PAS Avoidance Behavior:** significant adjusted difference favoring liraglutide (p <.05).  **Metabolic:**  **HbA1c:** −0.2% (liraglutide) vs +0.1% (placebo), p =.001.  **BMI:** −2.1 (liraglutide) vs −0.3 (placebo), p <.001.  **HOMA-IR:** +0.1 (liraglutide) vs +0.3 (placebo), p =.52; difference not significant.  **Safety:**  **Any AE:** 100% (liraglutide) vs 85.7% (placebo), p <.05.  **Most common AEs:** GI/appetite-related: nausea 57.1% vs 14.3% (p = 0.001); loss of appetite 61.9% vs 0% (p <.001). | Once-daily liraglutide was safe and generally well tolerated in non-/pre-diabetic PD, and improved non-motor symptom burden, ADLs, and QoL despite no clear benefit on off-state motor scores or global cognition. Findings support larger multicenter trials to confirm whether benefits reflect symptomatic improvement, broader disease modification, or both |
| Meissner etal., 2024 | Change from baseline to Month 12 in MDS-UPDRS Part III (on-medication) | MoCA | MDS-UPDRS Part II, PDQ-39, MDS-UPDRS Part I, MDS-UPDRS Part IV | Not assessed | **Motor:**  **MDS-UPDRS Part III, on-medication:** at 12 months, placebo worsened by 3.04 points (95% CI 1.46 to 4.62), while lixisenatide changed by −0.04 points (95% CI −1.62 to 1.54), giving a between-group difference of 3.08 points (95% CI 0.86 to 5.30; P=.007).  At 14 months, **Part III off-medication after 2-month washout:** mean scores were 17.7 (lixisenatide) vs 20.6 (placebo), difference 3.0 points (95% CI 0.1 to 5.8).  **Cognitive / nonmotor outcomes:**  No clear cognitive/nonmotor advantage was demonstrated in the reported secondary/exploratory results.  Difference between groups on MoCA score was -0.4 [-1.1, 0.2]  **Other outcomes (secondary/exploratory):**  At 12 months non-significant difference across MDS-UPDRS total, MDS-UPDRS Part I, MDS-UPDRS Part II, MDS-UPDRS Part IV, LEDD.  **Safety:**  **Any adverse event:** 86% with lixisenatide vs 71% with placebo. | Lixisenatide modestly but significantly reduced motor progression in early PD over 12 months, consistent with possible disease-modifying activity, but effects were largely limited to the primary motor endpoint and accompanied by frequent GI AEs. Larger, longer trials with biomarker confirmation are needed to establish true neuroprotective efficacy |
| McGarry, 2024 | Change from baseline to Week 36 in the sum of MDS-UPDRS Parts II + III | MoCA and SCOPA-Cog | MDS-UPDRS Part I, SE-ADL, PDQ-39 | DaT SPECT imaging | **Primary endpoint:**  In 255 early untreated PD participants, weekly NLY01 (2.5 mg or 5.0 mg) for 36 weeks did not improve MDS-UPDRS II+III vs placebo (2.5 mg: −0.39, 95% CI −2.96 to 2.18; p=.77; 5.0 mg: +0.36, −2.28 to 3.00; p=.79).  **Secondary:** No significant between-group differences across MDS-UPDRS Parts I–III, SE-ADL, PDQ-39, MoCA, SCOPA-Cog, NMSS, CGI-S/PGI-S.  **Safety:** Generally safe/tolerable; dose-related GI AEs and higher discontinuation in 5.0 mg arm; Weight loss AE was slightly more common with NLY01 (both doses 6%) vs placebo (2%).  **Exploratory:**  A prespecified age subgroup (<60 vs ≥60) suggested a more “beneficial” pattern on the primary endpoint in younger participants, but the authors emphasize this may be driven by an unusually large placebo deterioration in the younger subgroup and should be interpreted cautiously. | Weekly NLY01 (2.5/5.0 mg) was safe but did not provide clinically meaningful motor or non-motor benefit versus placebo over 36 weeks and does not support NLY01 as symptomatic or disease-modifying therapy at tested doses. An exploratory younger-age subgroup signal warrants caution and could reflect atypical placebo decline; future work may focus on earlier/younger cohorts and target engagement rather than broad unselected populations |
| Vijiaratnam etal., 2025 | Change from baseline to Week 96 in MDS-UPDRS Part III (off-medication) | MoCA | MDS-UPDRS Part I,  MDS-UPDRS Part II (ADL),  MDS-UPDRS Part III (on),  MDS-UPDRS Part IV,  Timed sit–stand–walk (off and on)  PDQ-39 summary index  EQ-5D-5L index + VAS  NMSS  PHQ-9  UDysRS  Hauser diaries (on/off time, dyskinesia)  LEDD change | DaT-SPECT, CSF α-syn seed amplification assay | **Motor:**  In 194 non-diabetic PD participants treated for 96 weeks, weekly exenatide 2 mg did not slow motor progression; MDS-UPDRS Part III (off) worsened similarly (adjusted difference 0.92, 95% CI −1.56 to 3.39; p=0.47).  **Secondary:**  No significant differences across multiple clinical, QoL, diary, and medication outcomes (including MoCA, NMSS, PDQ-39, EQ-5D-5L, UDysRS, PHQ-9, Hauser diaries, LEDD).  **Biomarkers:**  No effect on dopaminergic terminal loss on DaT-SPECT; no responsive subgroup including α-syn-positive CSF.  **Safety:**  More GI AEs, transient hyperamylasaemia ((9%) exenatide vs (1%) placebo).  **Serious AEs:** (9%) exenatide vs 11 (11%) placebo.  **Weight change:** –1.8 kg vs –1.3 kg (placebo); difference 0.5 kg. No relation to motor response. | Extended-release exenatide 2 mg weekly for 96 weeks was safe but showed no clinical or DaT-SPECT biomarker benefit, not supporting exenatide as disease-modifying therapy in PD population.  Authors recommend future work prioritize agents with stronger CNS target engagement and biologically defined subgroups (e.g., insulin-resistant phenotypes) rather than abandoning the GLP-1 pathway |
| Gejl et al.,2016 | Change from baseline in brain amyloid-β deposition | Wechsler Memory Scale IV | FAQ / ADCS-ADL, CDR, NPI, MRI volumetry | Amyloid PET – [¹¹C] PIB, FDG PET – CMRglc | **Amyloid PET ([¹¹C]PIB binding potential, BPND):**  Primary outcome was negative  **Within-group:** PIB binding increased in both groups:  **Placebo:** temporal lobe ratio increase 0.036 (95% CI 0.0012 to 0.070; p=.04).  **Liraglutide:** temporal lobe ratio increase 0.056 (95% CI 0.0017 to 0.11; p=.04) and occipital ratio increase 0.048 (95% CI 0.0013 to 0.094; p=.04).  **Between-group:** no significant differences in session 2/session 1 PIB ratios in any ROI (p ≥.38).  **Cognitive outcomes:**  **WMS-IV brief cognitive exam:**  **Total score:** no meaningful change and no between-group difference:  Change after 6 months: placebo −1.7 vs liraglutide −0.43, p=.50.  **Orientation subtest:** significant worsening within placebo (p=.041) but not in liraglutide; however, the between-group comparison for orientation change did not reach significance (p=.085).  **Neurodegeneration biomarker: cerebral glucose metabolism ([¹⁸F]FDG CMRglc):**  Placebo: significant CMRglc declines across multiple regions after 6 months, including:  Precuneus: −3.2 µmol/hg/min (95% CI 5.45 to 0.92), p=.009  Parietal: −2.1 (95% CI 4.21 to 0.081), p=.04  Temporal: −1.54 (95% CI 3.05 to 0.030), p=.046  Occipital: −2.10 (95% CI 3.61 to 0.59), p=.009  Cerebellum: −1.54 (95% CI 3.01 to 0.064), p=.04  **Liraglutide:** CMRglc showed numerical (non-significant) increases across regions (all p ≥.49).  **Between-group:** session 2/session 1 ratios in cingulate and occipital lobes differed significantly vs placebo (p=.04 for both), consistent with liraglutide preventing the metabolic decline seen in placebo.  **Surrogate cerebral blood flow (sCBF, derived from PIB early clearance):**  **Placebo:** significant sCBF increases (e.g., frontal +3.56 ml/hg/min, p=0.04; parietal +3.27, p=0.04; cerebellum +4.47, p=0.009; whole cortex +3.61, p=0.03).  **Liraglutide:** no significant sCBF changes (all p ≥ 0.41) and no significant between-group ratio differences (all p ≥ 0.13**).**  **Safety / metabolic:**  **Fasting glucose (6 months):** 5.6 mmol/L (placebo) vs 5.1 mmol/L (liraglutide), p=.0041; HbA1c unchanged.  **Weight loss (6 months):** 4.9 kg (liraglutide) vs 1.6 kg (placebo), (p<.01). Weight fell to 69.2 kg (liraglutide) vs 76.1 kg (placebo); change placebo vs change liraglutide p=.0083.  **BMI change also favored liraglutide** (change difference p=.0069).  **Blood pressure:** systolic and diastolic BP decreased significantly in liraglutide group by study end (systolic p=.015, diastolicp=.035; systolic change difference p=.013).  **Dropouts due to tolerability:** all dropouts occurred in liraglutide arm; one was drug-related (nausea/anorexia after 35 days). | Short-term liraglutide in established AD showed biological effects on cerebral glucose metabolism (maintenance of FDG-PET measures) but no effect on amyloid load or cognition, small sample size, baseline imbalances, and short duration limit definitive clinical inference. Authors conclude GLP-1 analogs remain promising, particularly if tested earlier in disease course and in larger/longer trials, but this study alone does not establish cognitive benefit in symptomatic AD |
| Gejl et al.,2017 post hoc study | Change from baseline in maximum blood–brain glucose transport capacity (Tmax) in cerebral cortex (FDG kinetic modeling with Michaelis–Menten analysis). |  | Glucose handling / PET kinetic measures |  | **Blood–brain barrier glucose transport capacity (Tmax):**  **Placebo:** no significant change in cortical Tmax over 6 months (p =.24, mean difference.093 µmol/g/min, 95% CI −0.037 to 0.22).  **Liraglutide:** cortical Tmax increased significantly after 6 months (p <.0001, mean difference.34 µmol/g/min, 95% CI 0.20 to 0.49).  **Between-group effect:** the increase in Tmax was significantly larger with liraglutide vs placebo (p =.0002, mean difference 0.25 µmol/g/min, 95% CI 0.13 to 0.37).  **Glucose handling / PET kinetic measures:**  **Net clearance (K and K):** no change with placebo (p =.53), but borderline increase with liraglutide (p =.049).  **Cerebral metabolic rate of glucose (CMRglc):** confirmed decline in placebo (p =.05) and no change in liraglutide (P =.58). | Post hoc findings suggest liraglutide may restore BBB glucose transport capacity (Tmax) toward healthy levels, consistent with a neurovascular mechanism potentially relevant to AD progression.  Correlations of Tmax/CMRglc with disease duration and cognition support the concept that impaired glucose transport contributes to progression; however, clinical implications are speculative due to post hoc design and small sample, requiring prospective confirmation in larger trials |
| Mullins etal., 2019 | Safety and tolerability | ADAS-Cog, MMSE, CDR-SB, and Digit Span (forward) | ADCS-ADL, CDR-sob (Global) | CSF Aβ42, total tau, p-tau181, MRI, MRS, EV Aβ42, EV Aβ40 | **Safety / tolerability:**  Exenatide was safe/tolerable in MCI and mild AD  GI tolerability signals (worse with exenatide):  **Nausea:** 38% (5/13) vs 0% (0/14), p=.016  **Upper GI upset (composite):** 62% vs 7%, p=.004  **Loss of appetite/weight loss:** 31% vs 0%, p=.041  **Clinical outcomes:**  Exenatide showed no meaningful benefit on cognition, daily function, MRI neurodegeneration measures, or core CSF biomarkers. **Exception:** There were Visit effects (changes over time in both groups) for CSF Aβ42 (Visit p=.008) and plasma Aβ40 (Visit p=.013). Extracellular vesicle showed a significant Group×Visit interaction (decreasing over time with exenatide vs placebo; p=.045), Better performance for exenatide sc. group in digit-span forward total score (p =.004).  **MRS:** A nominal Group×Visit signal for aspartate was reported (p=.024) but was strongly influenced by a placebo deviation at 6 months.  **Limitations**: Underpowered due to early termination. | This prematurely terminated Phase II study does not support a disease-modifying effect of exenatide in early AD; an isolated EV-derived Aβ42 change lacked accompanying cognitive/functional benefit with possible exception of improvement in attention and memory. Adequately powered trials would be required to clarify efficacy |
| Dei Cas et al., 2024 | Change from baseline to Week 32 in ADAS-Cog11 | ADAS-Cog11, MMSE, CDR, GDS, NPI, phonemic fluency | IADL, ADL (basic), CDR, NPI, GDS | Not assessed | **Cognition:**  In 32 non-diabetic MCI participants, 32 weeks of weekly long-acting exenatide 2 mg s.c. showed no overall improvement vs no-treatment control on ADAS-Cog11. **Effect modification:** Significant sex interaction driven by worsening in women on ADAS-Cog11 (p=.04).  **Secondary:** No benefit on MMSE, CDR, DS, NPI, or phonemic fluency over follow-up.  **Semantic fluency:** worsened with exenatide (significant treatment effect p=.04 and time×treatment p=.03).  **IADL:** mild worsening of instrumental ADLs with exenatide (significant time p=.02 and treatment p=.04).  These negative secondary effects were reported as more evident in females (IADL time effect p=.03; semantic fluency reductions also more evident).  **Metabolic**: Lowered fasting plasma glucose (p=.02) and body weight (p=.03); no change in HbA1c (p=.93) or incretin hormones.  **Safety:** More GI AEs (nausea/decreased appetite) with ~35% discontinuation; no serious AEs reported. | This small proof-of-concept trial does not support cognitive benefit of long-acting exenatide in non-diabetic MCI over 32 weeks and suggests a possible sex-specific harm signal (worsening in women).  Peripheral metabolic improvements appeared decoupled from cognition; due to limited sample size, short follow-up, and high dropout, conclusions are cautious, but results do not currently support incretin therapy as disease-modifying in MCI while motivating larger studies and investigation of sex-related heterogeneity |
| Watson etal., 2019 | Change from baseline in RSFC between bilateral hippocampus and default mode network regions | BVRT, CVLT-II (learning; short- and long-delay recall), D-KEFS (letter/category fluency; switching; Color-Word; Trails), Rey Complex figure (immediate/delayed recall), WAIS-III Digit Span and Digit Symbol, plus additional measures (e.g., Purdue Pegboard) | Only subjective cognitive complaints (MFQ) and depression scales | Resting-state fMRI (seed-based RSFC) with bilateral hippocampus | **Resting-state fMRI hippocampal connectivity (seed-based RSFC; baseline vs 12 weeks):**  **At baseline:** higher FPG was associated with decreased connectivity between the bilateral hippocampus and anterior medial frontal structures.  **At 12 weeks:** compared with placebo, the liraglutide group showed greater positive connectivity between the bilateral hippocampus and 3 clusters:   - Left middle frontal gyrus/frontal pole (incl. left superior/inferior frontal and paracingulate) - Posterior cingulate/precuneus - Left lateral occipital/parietal regions (incl. supramarginal/angular/postcentral)   These treatment-related increases were interpreted as increased intrinsic connectivity within DMN-related regions.  **Cognitive outcomes:**   - No detectable cognitive benefit: repeated-measures analyses showed no time × treatment effects on objective cognitive performance after 12 weeks. - Subjective cognition (MFQ): no changes within or between groups in subjective cognitive complaints.   **Other outcomes (metabolic / clinical):**  **FPG:** no baseline difference between groups.  **Glucose tolerance (OGTT 120 min):** declined slightly more in the liraglutide group over the study, but this was not statistically significant (p =.06).  **Attrition context:** dropout at time point 2 was mostly due to GI side effects or concerns about potential side effects. | In at-risk mid-life adults, liraglutide altered hippocampal–DMN functional connectivity without short-term cognitive benefit, suggesting neural network changes may precede detectable cognitive effects.  Larger and longer trials in higher-risk or cognitively impaired groups are needed to determine clinical relevance and potential neuroprotective benefit |
| Edison 2025 | Change from baseline in rCMRglc in the cortical regions (combination of regions:  hippocampal, medial temporal lobe and posterior cingulate) | ADAS-Executive z-score | CDR-SOB; ADCS-ADL z-score | FDG-PET rCMRglc (primary); MRI (hippocampus, temporal, parietal, whole GM, ventricles); TSPO-PET subgroup; plasma markers | **Change in [18F]FDG PET SUV at 52 weeks**:  Showed no significant between-group difference (adjusted difference −0.17, 95% CI −0.39 to 0.06, p=.14), and the PET spectral sensitivity analysis was also non-significant (difference −0.006, 95% CI −0.02 to 0.008, p=.38).  **Cognition:**  ADAS-Exec showed a small non-significant difference at 24 weeks (difference 0.07, 95% CI −0.05 to 0.19, p=.24), but a significant benefit at 52 weeks (difference 0.15, 95% CI 0.03 to 0.28, p=.01).  **Functional/global outcomes:**  Not significantly different at 24 or 52 weeks: CDR-SoB (24w: −0.01, 95% CI −0.51 to 0.50; 52w: −0.06, 95% CI −0.57 to 0.44) and ADCS-ADL (24w: −1.59, 95% CI −4.09 to 0.92; 52w: −0.58, 95% CI −3.13 to 1.97).  **MRI:**  There was no significant treatment difference in hippocampus/entorhinal or ventricular volumes (pre-specified MRI analyses), but exploratory analyses suggested less volume loss in the liraglutide group in the temporal lobe (696 mm³, 95% CI 184.37–1,208.12, p<.001) and total gray matter (7,274 mm³, 95% CI 2,704.05–11,844.8, p=.002), with additional signals/trends in parietal and frontoparietal lobes; exploratory VBM analyses also suggested slower reductions across several regions (e.g., frontal, parietal, temporal, whole cortical gray matter, and white matter) with the reported effect sizes and CIs. **Safety:**  There were 991 AEs total (placebo: 450 events in 87 participants; treatment: 541 events in 88 participants), and serious AEs occurred in 18 (17.6%) placebo vs 7 (6.9%) liraglutide participants; gastrointestinal events were most common, and weight loss was more frequent with liraglutide (e.g., up to 5% loss 39.2% vs 12.6%, and 5–10% loss 8.9% vs 1.1%). | Liraglutide did not improve the primary FDG-PET endpoint, but showed a modest cognitive signal on ADAS-Exec at 52 weeks and exploratory MRI signals suggesting less brain volume loss in some regions, with an overall clinically acceptable safety and tolerability profile in participants without diabetes or obesity, with secondary cognitive and MRI signals described as supportive for further confirmatory trials; definitive disease-modifying conclusions require larger Phase 3 evaluation |

AD: Alzheimer’s disease; ADAS-Cog: Alzheimer’s Disease Assessment Scale–Cognitive Subscale; ADAS-Cog11: ADAS-Cog (11-item version); ADAS-Cog13: ADAS-Cog (13-item version); ADAS-Exec: Alzheimer’s Disease Assessment Scale–Executive (composite/executive function score); ADCS-ADL: Alzheimer’s Disease Cooperative Study–Activities of Daily Living; ADL: activities of daily living (basic); AE: adverse event; AEs: adverse events; Aβ40: amyloid-β 40; Aβ42: amyloid-β 42; BMI: body mass index; BPND: binding potential (non-displaceable); BVRT: Benton Visual Retention Test; CDR: Clinical Dementia Rating; CDR-SB: Clinical Dementia Rating–Sum of Boxes; CI: confidence interval; CMRglc: cerebral metabolic rate of glucose; CNS: central nervous system; CSF: cerebrospinal fluid; CVLT-II: California Verbal Learning Test–Second Edition; DaT: dopamine transporter; DaT-SPECT: dopamine transporter single-photon emission computed tomography; DaTscan: [¹²³I]ioflupane dopamine-transporter SPECT scan; DAT: dopamine transporter; D-KEFS: Delis–Kaplan Executive Function System; DMN: default mode network; DRS-2: Dementia Rating Scale–2; DS: Digit Span; EQ-5D: EuroQol 5-Dimensions questionnaire; EQ-5D-5L: EuroQol 5-Dimensions 5-Level questionnaire; EV: extracellular vesicle(s); FAQ: Functional Activities Questionnaire; FDG: fluorodeoxyglucose; FDG-PET: fluorodeoxyglucose positron emission tomography; FPG: fasting plasma glucose; fMRI: functional magnetic resonance imaging; GDS: Geriatric Depression Scale; GI: gastrointestinal; GLP-1: glucagon-like peptide-1; GLP-1RA: glucagon-like peptide-1 receptor agonist; GM: gray matter; HbA1c: glycated hemoglobin; HOMA-IR: Homeostatic Model Assessment of Insulin Resistance; HR: hazard ratio; IADL: instrumental activities of daily living; K: kinetic rate constant/clearance parameter in FDG-PET kinetic modeling; LEDD: levodopa equivalent daily dose; MADRS: Montgomery–Åsberg Depression Rating Scale; MCI: mild cognitive impairment; MDS-UPDRS: Movement Disorder Society–Unified Parkinson’s Disease Rating Scale; MDRS-2: Mattis Dementia Rating Scale–2; MFQ: Memory Functioning Questionnaire; MMSE: Mini-Mental State Examination; MoCA: Montreal Cognitive Assessment; MRI: magnetic resonance imaging; MRS: magnetic resonance spectroscopy; NMSS: Non-Motor Symptoms Scale; NMS: non-motor symptoms; NMS Quest: Non-Motor Symptoms Questionnaire; NPI: Neuropsychiatric Inventory; NR: not reported; off: “off” medication state; OGTT: oral glucose tolerance test; on: “on” medication state; OR: odds ratio; p: p-value; PAS: Parkinson Anxiety Scale; PD: Parkinson’s disease; PDQ-39: Parkinson’s Disease Questionnaire–39; PET: positron emission tomography; pTau181: phosphorylated tau at threonine 181; QoL: quality of life; rCMRglc: regional cerebral metabolic rate of glucose; RCT: randomized controlled trial; ROI: region of interest; RSFC: resting-state functional connectivity; s.c.: subcutaneous; sCBF: surrogate cerebral blood flow; SCOPA: Scales for Outcomes in Parkinson’s Disease; SCOPA-Cog: SCOPA–Cognition; SE-ADL: Schwab & England Activities of Daily Living scale; SPECT: single-photon emission computed tomography; SUV: standardized uptake value; Tmax: maximum transport capacity; TSPO: translocator protein; TSPO-PET: translocator protein PET; UDysRS: Unified Dyskinesia Rating Scale; VAS: visual analog scale; VBM: voxel-based morphometry; WAIS-III: Wechsler Adult Intelligence Scale–Third Edition; WMS-IV: Wechsler Memory Scale–Fourth Edition; [¹¹C]PIB: carbon-11 Pittsburgh Compound B (amyloid PET tracer); [¹²³I]FP-CIT: iodine-123 FP-CIT (ioflupane; dopamine-transporter SPECT tracer); [¹⁸F]FDG: fluorine-18 fluorodeoxyglucose (PET tracer).

# **Table S9.** Non-diabetic status definition, follow up time, and funding information in the included trials

| **Study ID** | **Non-diabetic status definition** | **Follow up** | **Funding / sponsor** |
| --- | --- | --- | --- |
| Gejl et al.,2016 | Diabetes mellitus was an exclusion criterion | Baseline and Month 6 | Novo Nordisk Scandinavia, Aarhus University |
| Mullins etal., 2019 | Diabetes excluded based on fasting plasma glucose and oral glucose tolerance test | Baseline, Week 1, Week 2, Month 6, Month 12, and Month 18 | Intramural Research Program of the National Institute on Aging, AstraZeneca |
| Watson etal., 2019 | Type 2 diabetes excluded using fasting plasma glucose and 2-hour oral glucose tolerance test | Baseline and Week 12 | American Diabetes Association |
| Dei Cas et al., 2024 | Diabetes mellitus excluded according to American Diabetes Association criteria | Baseline, Week 16, and Week 32 | Diabetes Research Innovation (2015), University of Parma |
| Edison 2025 | All participants with diabetes excluded; use of diabetes medications not permitted | Safety visits: Baseline, Week 1, Week 4, Week 8, Week 12, Week 16, Week 20, Week 24, Week 28, Week 32, Week 36, Week 40, Week 44, Week 48, Week 52, and Week 56; [18F] FDG and MRI scans: Baseline and Week 52; ADAS-Exec, CDR-SoB and ADCS-ADL: Baseline, Week 24 and Week 52 | Alzheimer’s Society, Alzheimer’s Drug Discovery Foundation,Van Geest Foundation, NIHR Imperial Biomedical Research Centre, Novo Nordisk A/S |
| Aviles-Olmos et al., 2013 | Diabetes mellitus was an exclusion criterion | Baseline, Month 6, Month 12, and Month 14 | Cure Parkinson’s Trust, University College London |
| Athauda et al., 2017 | Diabetes excluded at screening (HbA1c ≥48 mmol/mol) | Baseline, Week 12, Week 24, Week 36, Week 48, and Week 60 | Michael J Fox Foundation for Parkinson’s Research |
| Hogg etal., 2022 | Excluded participants with current diabetes or use of antidiabetic medications | Safety/monitoring: Week 4, Week 12, and Week 38; full clinical outcome assessments: Week 26 and Week 52 (after titration | Cure Parkinson’s, liraglutide supplied by Novo Nordisk |
| McGarry, 2024 | Current diagnosis of diabetes was an exclusion criterion | Safety follow-ups: Week 40 and Week 44; clinical assessments: Week 4, Week 12, Week 24, Week 36) | D&D Pharmatech (Neuraly) |
| Meissner etal., 2024 | Type 1 and type 2 diabetes explicitly excluded | Baseline, Day 15, Month 1, Month 3, Month 6, Month 9, Month 12, and Month 14 | French Ministry of Health and Cure Parkinson’s, Sanofi |
| Vijiaratnam etal., 2025 | Type 1 or type 2 diabetes explicitly excluded | Baseline, Week 24, Week 48, Week 72, and Week 96 | National Institute for Health and Care Research EME scheme, Cure. Parkinson’s, AstraZeneca, University college London |

ADAS-Exec, Alzheimer’s Disease Assessment Scale–Executive Function; ADCS-ADL, Alzheimer’s Disease Cooperative Study–Activities of Daily Living; CDR-SoB, Clinical Dementia Rating–Sum of Boxes; FDG, fluorodeoxyglucose; HbA1c, glycated hemoglobin; MRI, magnetic resonance imaging; NIHR, National Institute for Health and Care Research.

# **
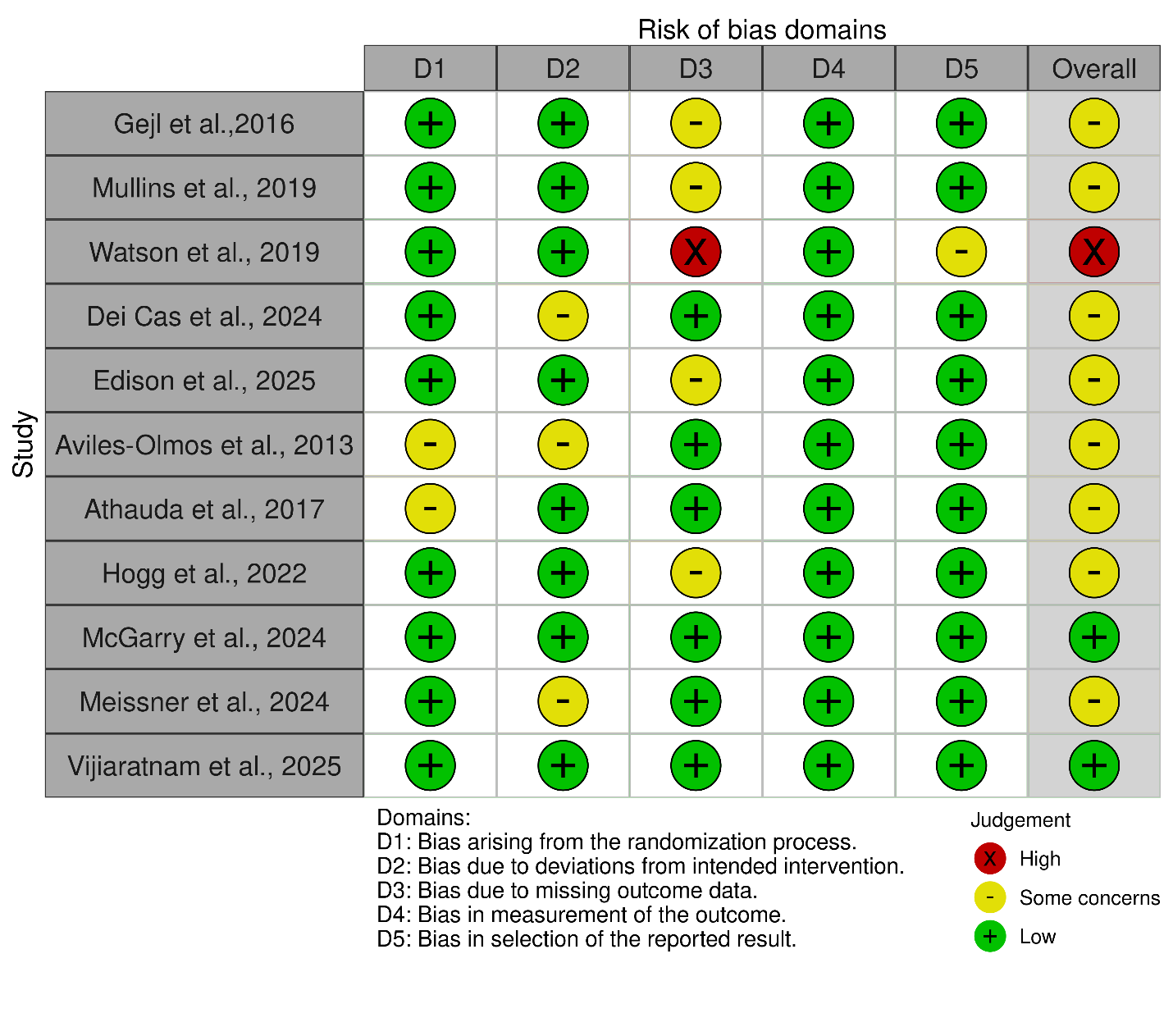
****Fig. S1** Risk of bias assessment of included trials.

# **
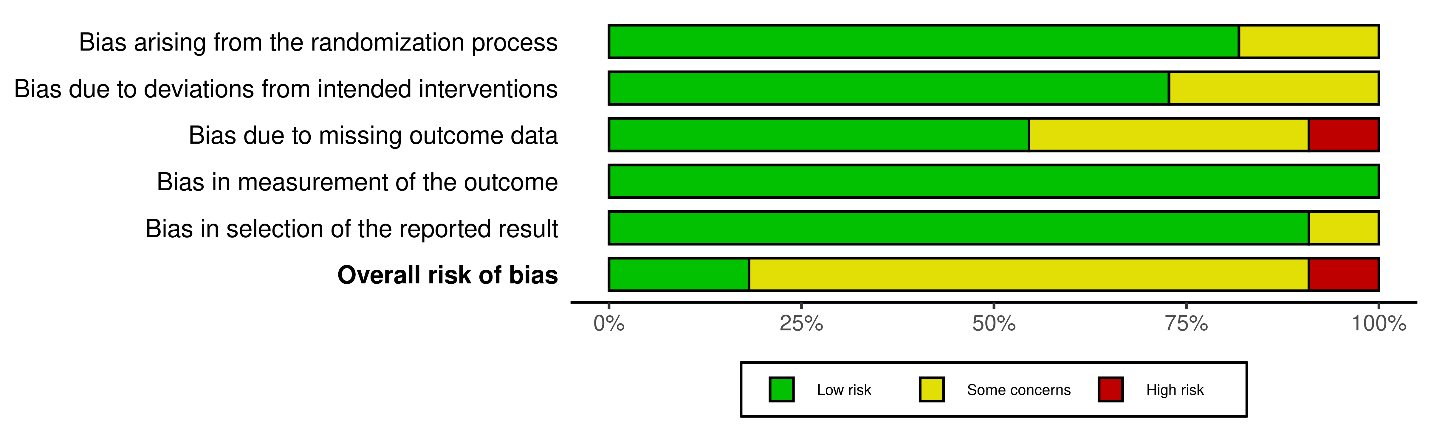
****Fig. S2** Summary plot of risk of bias assessment.

# **
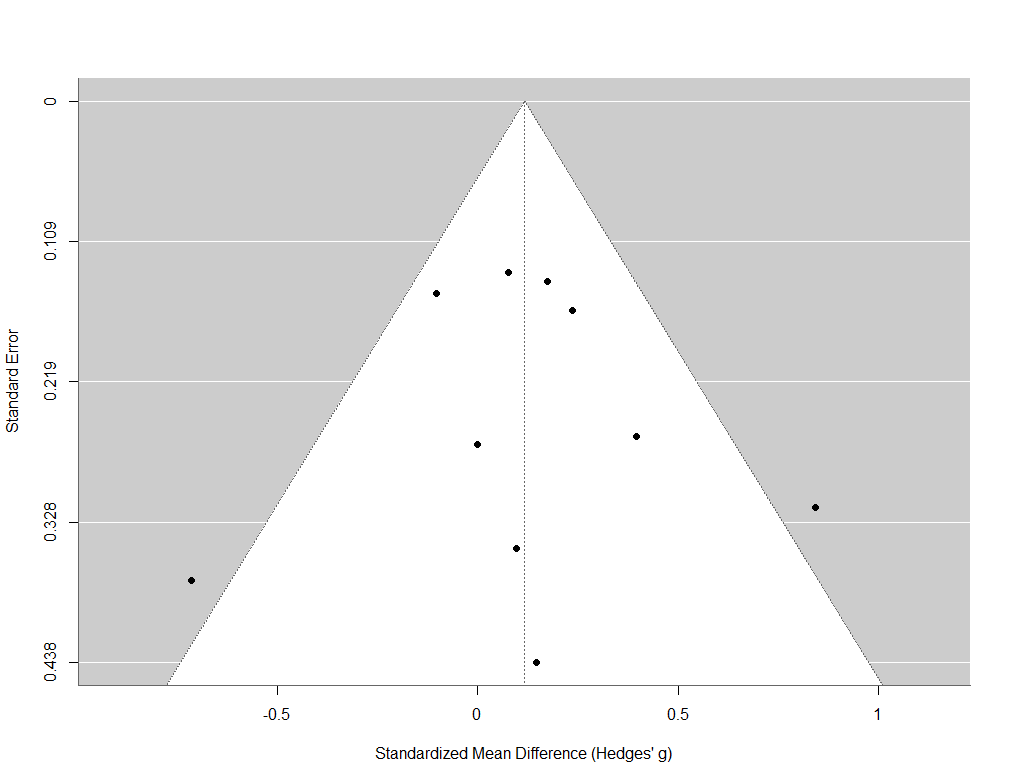
Fig. S3** Funnel plot for the global cognition outcome. The plot displays study-specific effect estimates against their standard errors to assess possible small-study effects or publication bias. In the absence of such bias, the studies are expected to be distributed symmetrically around the pooled effect estimate.

# **Fig. S4** Sensitivity analyses for global cognition. (a) Sensitivity analysis using alternative global cognitive measures when multiple scales were reported within a trial. (b) Sensitivity analysis excluding the Dei Cas et al., 2024 trial (MCI/prediabetic-enrolling study). **Note:** Dei Cas et al., 2024, Mullins et al., 2019, and McGarry et al., 2024 reported multiple cognitive instruments; the primary global cognitive outcome was selected according to the prespecified hierarchy. Effect estimates are presented as SMDs with 95% CIs. Positive SMD values indicate better global cognitive performance with GLP-1RAs than with control. CI, confidence interval; GLP-1RA, glucagon-like peptide-1 receptor agonist; MCI, mild cognitive impairment; SMD, standardized mean difference.

# **
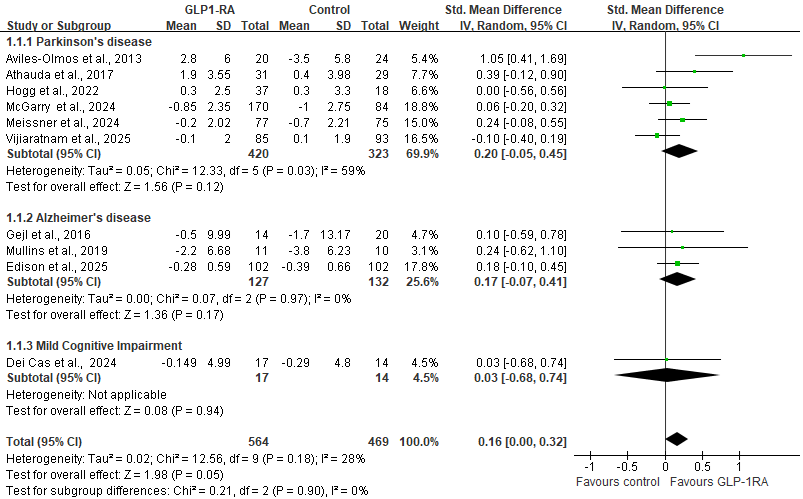
Fig. S5** Sensitivity analyses for global cognition: end-of-treatment effects across trials, with post-treatment washout cognition available only in two PD trials (Avilés-Olmos et al., 2013; Athauda et al., 2017). Effect estimates are presented as SMDs with 95% CIs. Positive SMD values indicate better global cognitive performance with GLP-1RAs than with control. GLP-1RA, glucagon-like peptide-1 receptor agonist; SMD, standardized mean difference; CI, confidence interval.

**Fig. S6** Pooled mean differences in cognitive outcomes and MCID-based interpretation of MMSE and ADAS-Cog. (a) Cognitive outcome assessed by MDRS-2. (b) Cognitive outcome assessed by MoCA. (c) Cognitive outcome assessed by MMSE. (d) Cognitive outcome assessed by ADAS-Cog. (e) MCID-based interpretation of the pooled effect on MMSE. (f) MCID-based interpretation of the pooled effect on ADAS-Cog. For MMSE, MDRS-2, and MoCA, positive MDs favor GLP-1RAs because higher scores indicate better cognition. For ADAS-Cog, negative MDs favor GLP-1RA because higher scores indicate worse cognition. MMSE and ADAS-Cog were evaluated in MCI and AD populations, whereas MDRS-2 and MoCA were evaluated in PD populations. AD, Alzheimer’s disease; ADAS-Cog, Alzheimer’s Disease Assessment Scale–Cognitive Subscale; CI, confidence interval; GLP-1RA, glucagon-like peptide-1 receptor agonist; MCID, minimal clinically important difference; MCI, mild cognitive impairment; MD, mean difference; MDRS-2, Mattis Dementia Rating Scale–Second Edition; MMSE, Mini-Mental State Examination; MoCA, Montreal Cognitive Assessment; PD, Parkinson’s disease.

# **Fig. S7** Effects of GLP-1 receptor agonists (GLP-1RAs) on domain-specific cognitive outcomes. Forest plots present standardized mean differences (SMDs) with 95% confidence intervals (CIs) comparing GLP-1RAs with control. Cognitive domains include (a) verbal fluency, (b) executive function (set-shifting), (c) verbal learning and memory, (d) attention/working memory, and (e) visual memory. Positive SMD values favor GLP-1RA treatment.

# **Fig. S8** Effects of GLP-1 receptor agonists (GLP-1RAs) on functional, clinical severity, and depression outcomes. Forest plots show standardized mean differences (SMDs) with 95% confidence intervals (CIs). (a) Functional outcomes, (b) clinical severity outcomes, and (c) depressive symptoms. Negative SMD values favor GLP-1RA treatment.

# **
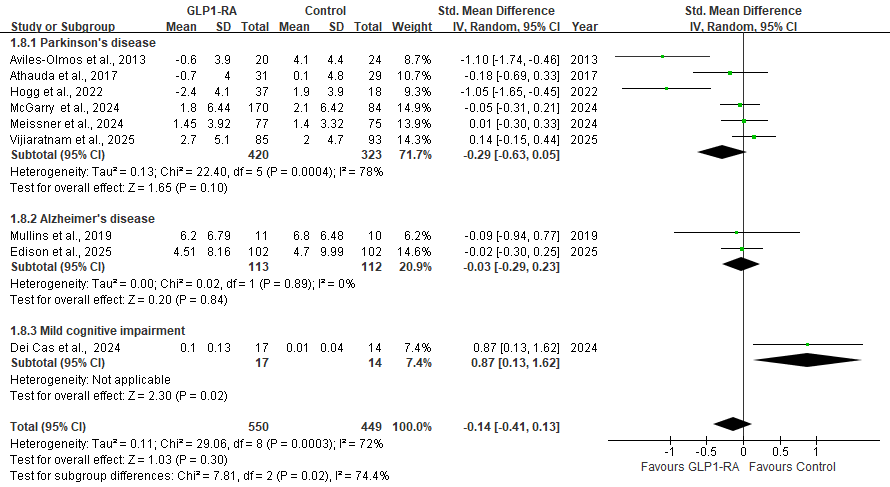
** **Fig. S9** Sensitivity analysis of functional outcomes using alternative functional measures when multiple scales were reported within a trial. Forest plots show standardized mean differences (SMDs) with 95% confidence intervals (CIs). Negative SMD values favor GLP-1 receptor agonist (GLP-1RA) treatment. **Note:** McGarry et al., 2024 only reported multiple functional scales.

**Fig. S10** Pooled mean differences in CDR-SoB and MADRS outcomes and MCID-based interpretation. (a) Functional outcome assessed by CDR-SoB. (b) Depression outcome assessed by MADRS. (c) MCID-based interpretation of the pooled effect on CDR-SoB. (d) MCID-based interpretation of the pooled effect on MADRS. For CDR-SoB and MADRS, negative MDs favor GLP-1RA because higher scores indicate worse clinical status. CDR-SoB was evaluated in AD populations, whereas MADRS was evaluated in PD populations. AD, Alzheimer’s disease; CDR-SoB, Clinical Dementia Rating–Sum of Boxes; CI, confidence interval; GLP-1RA, glucagon-like peptide-1 receptor agonist; MADRS, Montgomery–Åsberg Depression Rating Scale; MCID, minimal clinically important difference; MCI, mild cognitive impairment; MD, mean difference; PD, Parkinson’s disease.

**Fig. S11** MCID-based interpretation of pooled mean differences in Parkinson’s disease–related outcomes. (a) quality of life assessed by PDQ-39 SI, (b) non-motor experiences of daily living assessed by MDS-UPDRS Part I, (c) motor experiences of daily living assessed by MDS-UPDRS Part II, (d) motor examinations off medication assessed by MDS-UPDRS Part III, (e) motor examinations on medication assessed by MDS-UPDRS Part III, and (f) motor complications assessed by MDS-UPDRS Part IV. To read the graphs, the black dot and horizontal line represent the pooled MD and its 95% CI, the vertical dashed lines indicate the published MCID thresholds for improvement and decline, and the shaded areas under each curve represent the probabilities of any improvement, clinically important improvement, any decline, and clinically important decline relative to those thresholds. Lower values indicate greater improvement across these outcomes. CI, confidence interval; MCID, minimal clinically important difference; MD, mean difference; MDS-UPDRS, Movement Disorder Society–Unified Parkinson’s Disease Rating Scale; PDQ-39 SI, Parkinson’s Disease Questionnaire-39 Summary Index.

**
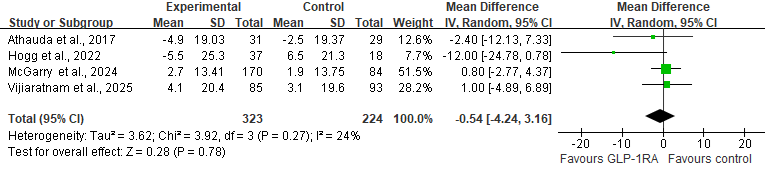
**

# **Fig. S12** Effects of GLP-1 receptor agonists on non-motor symptoms measured using a non-motor symptom scale (NMSS). Forest plots show mean differences (MDs) with 95% confidence intervals (CIs). Lower MDs favor GLP-1RA because higher scores indicate worse clinical status.


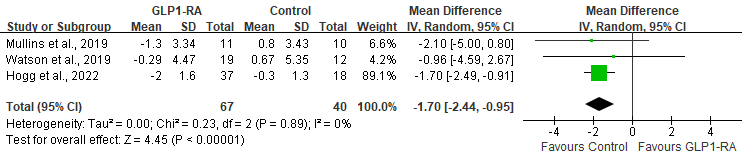


# **Fig. S13** Effects of GLP-1 receptor agonists on body mass index. Forest plots show mean differences with 95% confidence intervals (CIs) comparing GLP-1 receptor agonists with control. Values below 0 indicate lower body mass index with GLP-1 receptor agonist group, whereas values above 0 indicate higher body mass index. The vertical line at 0 indicates no difference between groups.

# **
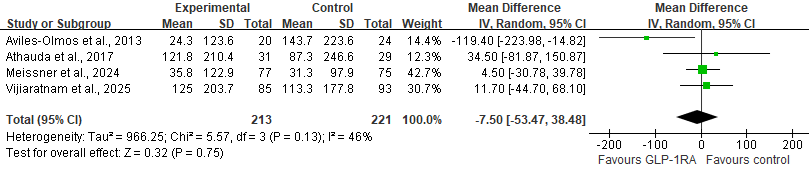
****Fig. S14** Effects of GLP-1 receptor agonists (GLP-1RAs) on levodopa equivalent daily dose. Forest plot of pooled mean differences with 95% confidence intervals (CIs) comparing GLP-1RAs with control. Values below 0 indicate a lower levodopa equivalent daily dose with GLP-1RAs treatment, whereas values above 0 indicate a higher dose. The vertical line at 0 indicates no difference between groups.


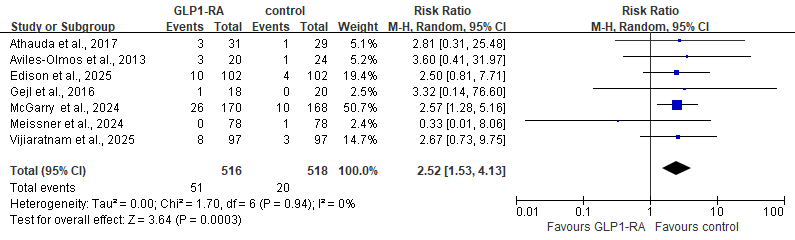


**Fig. S15** Effect of GLP-1 receptor agonists (GLP-1RAs) on discontinuation due to adverse events. Forest plot showing the pooled risk ratio with 95% confidence intervals (CIs) for treatment discontinuation due to adverse events in trials comparing GLP-1RAs with control. Values greater than 1 indicate more discontinuations with GLP-1RAs group.

**Fig. S16** Effects of GLP-1 receptor agonists (GLP-1RAs) on selected safety outcomes. Forest plots show pooled risk ratios with 95% confidence intervals (CIs) for (a) headache, (b) dizziness, (c) fatigue, (d) anxiety, (e) fall, (f) decreased appetite, (g) weight loss, and (h) hypoglycemia, comparing GLP-1RAs versus control. Values greater than 1 indicate a higher risk with GLP-1RA group.

**Fig. S17** Effects of GLP-1 receptor agonists (GLP-1RAs) on gastrointestinal adverse events. Forest plots show pooled risk ratios with 95% confidence intervals (CIs) for (a) nausea, (b) vomiting, (c) diarrhea, (d) constipation, (e) abdominal pain, and (f) dyspepsia, comparing GLP-1RAs versus control. Values greater than 1 indicate a higher risk with GLP-1RA group.


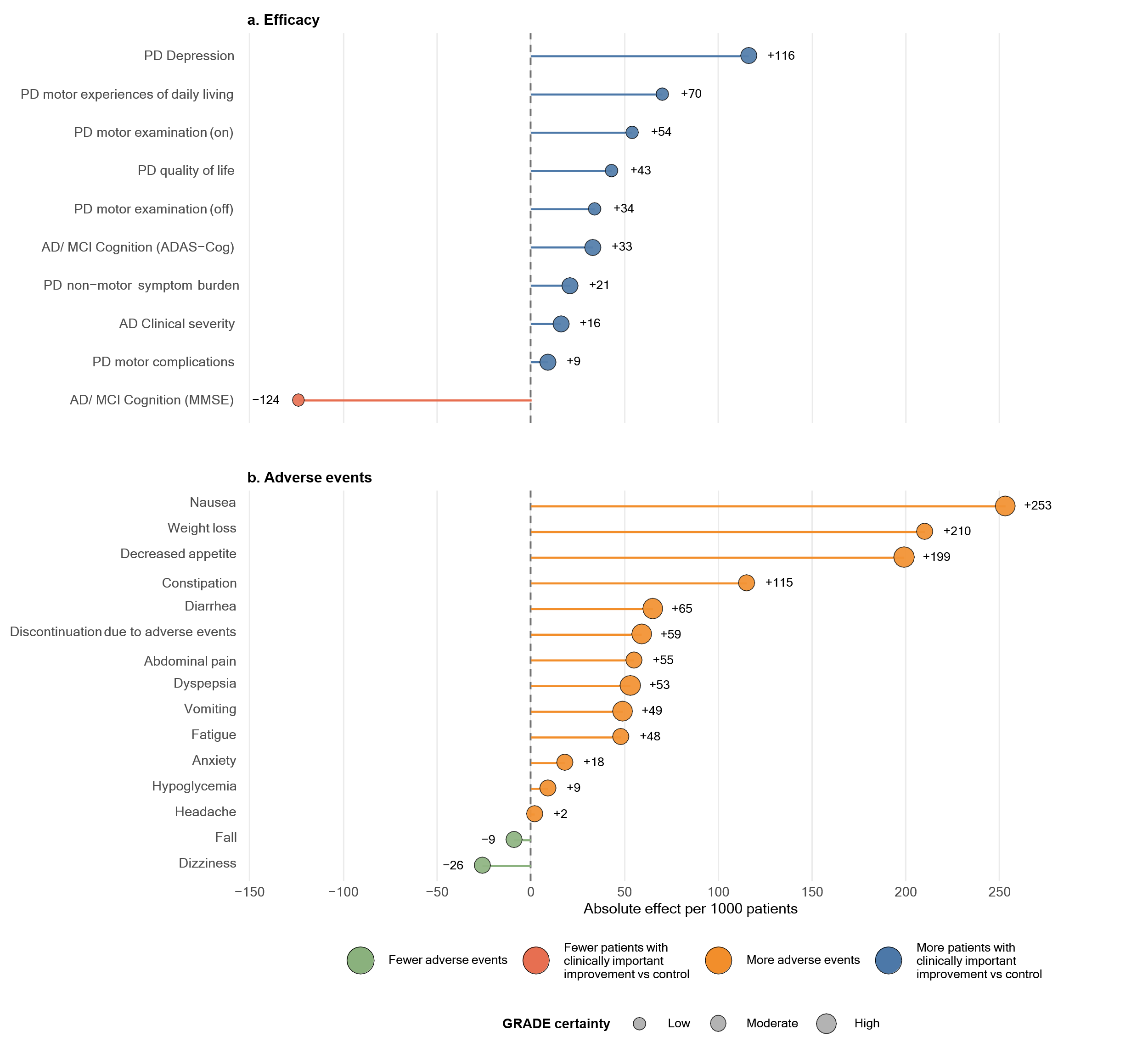


**Fig. S18** Risk–benefit profile of GLP-1RAs across efficacy and safety outcomes. (a) Efficacy. (b) Adverse events. Bubble plot showing absolute effects per 1000 patients for efficacy and adverse-event outcomes, together with certainty of evidence. Positive values indicate more adverse events or more patients achieving clinically important improvement versus control, whereas negative values indicate fewer adverse events or fewer patients achieving clinically important improvement versus control. Bubble size reflects GRADE certainty of evidence. **Note:** The risk with GLP-1RA (and its 95% CI) was calculated from the assumed risk in the control group and the relative effect of the intervention (and its 95% CI). For efficacy outcomes, absolute effects were estimated using the prespecified threshold for the minimal clinically important difference. CI, confidence interval; GLP-1RA, glucagon-like peptide-1 receptor agonist; GRADE, Grading of Recommendations, Assessment, Development and Evaluation.


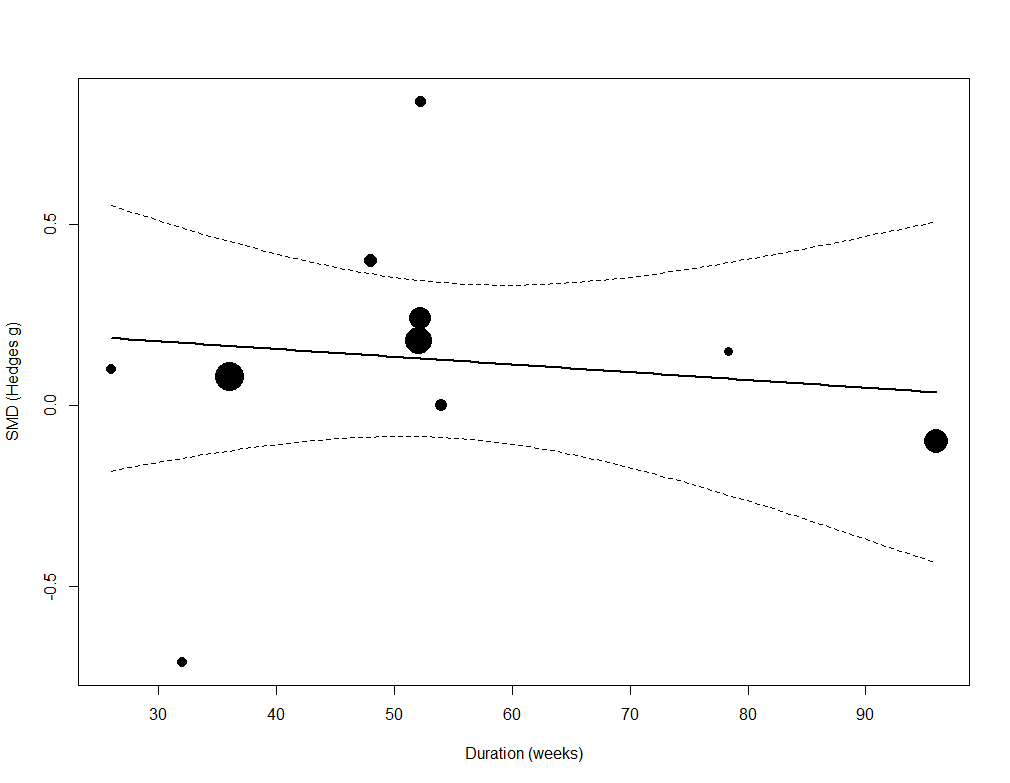


# **Fig. S19** Meta-regression analysis of the primary outcome.
